# Qing-Wen-Jie-Re Mixture Ameliorates Poly (I:C)-Induced Viral Pneumonia Through Regulating the Inflammatory Response and Serum Metabolism

**DOI:** 10.3389/fphar.2022.891851

**Published:** 2022-06-15

**Authors:** Qin Li, Tingrui Zhang, Yuming Wang, Shangsong Yang, Junyu Luo, Fang Fang, Jiabao Liao, Weibo Wen, Huantian Cui, Hongcai Shang

**Affiliations:** ^1^ Dongzhimen Hospital, Beijing University of Chinese Medicine, Beijing, China; ^2^ Postdoctoral Research Station, Yunnan Provincial Hospital of Traditional Chinese Medicine, Kunming, China; ^3^ School of Basic Medical Sciences, Yunnan University of Traditional Chinese Medicine, Kunming, China; ^4^ The First School of Clinical Medicine, Yunnan University of Chinese Medicine, Kunming, China; ^5^ Graduate School, Tianjin University of Traditional Chinese Medicine, Tianjin, China; ^6^ Department of Emergency, Jiaxing Hospital of Traditional Chinese Medicine, Jiaxing, China; ^7^ School of Life Sciences, Shandong University, Qingdao, China

**Keywords:** viral pneumonia, Qing-Wen-Jie-Re Mixture, therapeutic effects, inflammatory response, metabolic modulatory

## Abstract

Qing-Wen-Jie-Re mixture (QWJR) has been used in the treatment of the coronavirus disease 2019 (COVID-19) in China. However, the protective mechanisms of QWJR on viral pneumonia remain unclear. In the present study, we first investigated the therapeutic effects of QWJR on a rat viral pneumonia model established by using polyinosinic-polycytidylic acid (poly (I:C)). The results indicated that QWJR could relieve the destruction of alveolar-capillary barrier in viral pneumonia rats, as represented by the decreased wet/dry weight (W/D) ratio in lung, total cell count and total protein concentration in bronchoalveolar lavage fluid (BALF). Besides, QWJR could also down-regulate the expression of inflammatory factors such as tumor necrosis factor-alpha (TNF-α), interleukin (IL)-1β and IL-6. More M1-type macrophage polarization was detected by calculating CD86^+^ cells and CD206^+^ cells and validated by the decline of inducible nitric oxide synthase (iNOS) and elevated arginase-1 (Arg-1) in lung. Finally, serum untargeted metabolomics analysis demonstrated that QWJR might take effect through regulating arginine metabolism, arachidonic acid (AA) metabolism, tricarboxylic acid (TCA) cycle, nicotinate and nicotinamide metabolism processes.

## 1 Introduction

In the first 20 years of the 21st century, regional to worldwide epidemics caused by respiratory viruses have occurred frequently (Gómez-Rial et al., 2020). Such as the severe acute respiratory syndrome coronavirus in 2003, the avian influenza in 2005, the influenza A H1N1 virus in 2009, the Middle East respiratory syndrome coronavirus in 2015, the H7N9 avian influenza virus in 2013, and the new coronavirus from 2019 to the present (Zhang et al., 2016; Swapnarekha et al., 2020). The production and life order worldwide were greatly affected during each pandemic (Liu et al., 2020). While viral characteristics such as high variability and complex pathogenic mechanisms severely constrained the development of vaccines and anti-viral drugs. Studies on developing safe and effective treatments for emerging respiratory infectious diseases are encouraged.

Traditional Chinese medicine (TCM) has been proved to have significant therapeutic effects on viral pneumonia (Wei et al., 2019; Chai et al., 2022). As a typical representative, Lian-Hua-Qing-Wen capsules with broad-spectrum anti-viral and immunomodulatory effects were used to treat many types of influenza (Tao et al., 2013; Ding et al., 2017). A combination of Hua-Shi-Bai-Du granules with conventional treatment could shorten the average cure time of COVID-19 patients (Li Q. et al., 2020; Cui et al., 2020). Hou-Yan-Qing could decrease the viral load in lung and has therapeutic effects on influenza A virus-infected mice (Wei et al., 2021). Xue-Bi-Jing injection has been used to treat influenza A virus subtype H1N1, human H7N9 influenza, Middle East respiratory syndrome coronavirus, Ebola virus and dengue fever virus (Tong et al., 2020), and a recent study found that Xue-Bi-Jing injection could reduce c-reactive protein expression, slow down erythrocyte sedimentation rate and increase white blood cell count in patients with severe COVID-19 (Wen et al., 2020). Hence, investigating the mechanism of TCMs in the treatment of viral pneumonia can provide a new strategy for developing new anti-respiratory virus drugs.

Qing-Wen-Jie-Re mixture (QWJR) has been proved to have a wide range of anti-viral and anti-inflammatory abilities against common coronavirus and emerging coronaviruses (Xie et al., 2021). Since the end of 2019, QWJR has been used to treat COVID-19 in Yunnan Province of China, Myanmar and Laos, and has proved to have satisfactory therapeutic effects. However, the mechanism of QWJR in anti-viral pneumonia remains unclear.

Viral infection will cause excessive activation of immune cells, such as macrophages and neutrophils, migrating into the lung tissue (Nicholls et al., 2003; Channappanavar et al., 2016) and producing large amounts of inflammatory factors, such as tumor necrosis factor-alpha (TNF-α), interleukin (IL)-1, and IL-6 (Li H. et al., 2020; Jose and Manuel, 2020), which is known as cytokine storm (Huang et al., 2020). Then the excessive immune response triggers diffuse damage to lung cells, leading to pulmonary fibrosis and even multi-organ damage (Gibson et al., 2020; Lamers et al., 2020; Neurath, 2020).

In recent years, many metabolites screened by untargeted metabolomics have provided an important theoretical basis for diagnosing and treating viral pneumonia. An independent study reported significant differences in the serum metabolic profiles at different stages of COVID-19, and the tricarboxylic acid (TCA) cycle and urea cycle may be involved in the pathological processes (Jia et al., 2021). Likewise, Jia et al. found the levels of IL-1β, TNF-α and IL-6 increased with the severity of COVID-19, and TCA cycle-related metabolites such as aspartate, creatine, malate and alpha-ketoglutaric acid (α-KG) were positively correlated with the expression of IL-1β, TNF-α and IL-6 (Jia et al., 2021). In an H1N1 (PR8) infectious mouse model, altered metabolic processes are closely related to the development of influenzas, such as serum tryptophan metabolism, fatty acid β-oxidation, sphingolipid metabolism, purine metabolism, and the biosynthesis of pantothenic acid and coenzyme A (Cui et al., 2016).

This study generated a viral pneumonia rat model using the intratracheal injection of poly(I:C) as a viral mimetic (Cui et al., 2020). The therapeutic effects of QWJR on viral pneumonia were investigated in the rat model, and the anti-inflammatory effects of QWJR on viral pneumonia were evaluated. Untargeted metabolomics techniques were used to investigate the metabolic regulatory mechanism of QWJR on viral pneumonia.

## 2 Materials and Methods

### 2.1 Reagents

Dexamethasone was obtained from Beijing Solarbia Technology Co., Ltd. (Beijing, China). IL-6, IL-1β, TNF-α enzyme-linked immunosorbent assay (ELISA) and arginase-1 (Arg-1) ELISA kit was obtained from Shanghai Enzymatic Union Biotechnology Co., Ltd. (Shanghai, China). Inducible nitric oxide synthase (iNOS) and bicinchoninic acid (BCA) protein concentration assay kits were purchased from Nanjing Jiancheng Biological Engineering Institute (Nanjing, China). CD86 polyclonal antibody and CD206 polyclonal antibody were purchased from Proteintech Group, Inc. (Wuhang, China). Poly(I:C) and reference standards of chlorogenic acid, forsythoside A, liquiritin, saikosaponin A, baicalin, arginine, magnolol, protocatechuic acid, 4-hydroxyacetophenone, aristolochic acid A and artemisinin were obtained from Shanghai Yuanye Biotechnology Co., Ltd. (Shanghai, China). All herbs used to prepare QWJR were obtained from Sinopharm Yunnan, Ltd. (Kunming, China).

### 2.2 Preparation and Characterization of QWJR

The production and clinical application of QWJR were approved by the local drug administration as shown in Supplementary Figure S1. The details of herbs used to prepare QWJR are listed in Table 1. According to the preparation standard for the medical institution in Yunnan (Approval number: Z2020006A), 15 g of *Pogostemon cablin* (Blanco) Benth. (Batch number: 20201601), 20 g of *Bupleurum candollei* Wall. ex DC. (Batch number: 20200412), 12 g of *Scutellaria amoena* C.H.Wright (Batch number: 20201520), 12 g of *Forsythia suspensa* (Thunb.) Vahl (Batch number: 20191803), 15 g of *Pinellia ternata* (Thunb.) Makino (Batch number: 20191339), 12 g of *Magnolia officinalis* Rehder & E.H.Wilson (Batch number: 20201114), 10 g of *Hedychium spicatum* Sm. (Batch number: 20191801), 15 g of *Vincetoxicum atratum* (Bunge) C.Morren & Decne. (Batch number: 20190126), 15 g of *Artemisia capillaris* Thunb. (Batch number: 20191411), 15 g of *Valeriana jatamansi* Jones ex Roxb. (Batch number: 20190715), 15 g of *Talcum* (Batch number: 20200302), 20 g of *Massa medicata fermentata* (Batch number: 20200801), and 9 g of *Glycyrrhiza uralensis* Fisch. ex DC. (Batch number: 20200111), a total of 13 herbs weighed 185 g were decocted three times: for the first decoction, 1,850 ml fresh water was added and decocted for 60 min before filtering; for the second decoction, 1,480 ml fresh water was added and decocted for 40 min before filtering; for the third decoction, 1,110 ml fresh water was added and decocted for 30 min before filtering. Finally, the filtrate was concentrated and adjusted to 300 ml with purified water, which means the specification of QWJR was 0.60 g (herbs)/mL (QWJR mixture).

**TABLE 1:** The details of herbs of QWJR.

Drug name	Plant part	Species name	Dosage (g)	Batch number
Herba pogostemonis	The dry aboveground part of *Pogostemon cablin* (Blanco) Benth	Lamiaceae	15	20201601
Radix bupleuri	The herb of *Bupleurum candollei* Wall. ex DC.	Umbelliferae	20	20200412
Scutellaria baicalensis georgi	The root or stem of *Scutellaria amoena* C.H.Wright	Lamiaceae	12	20201520
Fructus forsythiae	The dried fruit of *Forsythia suspensa* (Thunb.) Vahl	Oleaceae	12	20191803
Rhizome pinelliae	The dry tubers of *Pinellia ternata* (Thunb.) Makino	Araceae	15	20191339
Cortex magnoliae officinalis	The dried bark of *Magnolia officinalis* Rehder & E.H.Wilson	Magnoliaceae	12	20201114
Tsaoko amomum fruit	The dried fruit of *Hedychium spicatum* Sm	Zingiberaceae	10	20191801
Radix cynanchi atrati	The dried root of *Vincetoxicum atratum* (Bunge) C.Morren & Decne	Asclepiadaceae	15	20190126
Scopariae artemisiae herba	The herb of *Artemisia capillaris* Thunb	Compositae	15	20191411
Valeriana jatamansi jones	The dried root of *Valeriana jatamansi* Jones ex Roxb	Valerianaceae	15	20190715
Talcum	—	—	15	20200302
Massa medicata fermentata	—	—	20	20200801
Radix glycyrrhizae	The dried root of *Glycyrrhiza uralensis* Fisch. ex DC*.*	Leguminosae	9	20200111

For the characterization of QWJR, a total of 11 principal components in QWJR, including chlorogenic acid, forsythoside A, liquiritin, saikosaponin A, baicalin, arginine, magnolol, protocatechuic acid, 4-hydroxyacetophenone, aristolochic acid A, and artemisinin were used as the reference standards according to the primary chemical component detection in Pharmacopoeia of People’s Republic of China (2020 edition) issued by Chinese Pharmacopoeia Commission (Hu et al., 2021). Briefly, 5 mg of each reference standard was dissolved in 5 ml methanol and incubated at 50°C for 30 min to obtain the stock solution of each reference standard (1 mg/ml). Then, 100 μL of stock solution was dissolved in 900 μl methanol to get the test solutions of reference standards. Besides, 300 μl of QWJR were fully dissolved in 900 μl methanol and centrifuged at 13,000 rpm for 10 min and the supernatant was collected to obtain the test solution of QWJR.

An ACQUITY ultra-performance liquid chromatography (UPLC; Waters Corp, United States) coupled with Xevo G2 quadrupole-time-of-flight (Q-TOF) mass spectrometer (MS; Waters Corp., Milford, MA, United States) systems were used for the main chemical component detection of QWJR. Briefly, 2 μl of test solution was injected onto an ACQUITY UPLC BEH C18 Column (2.1 × 100 mm, 1.7 μm; column temperature: 50°C; flow rate: 0.3 ml/min). Mobile phase A was 0.1% formic acid aqueous solution and mobile phase B was acetonitrile containing 0.1% formic acid. The mobile phase conditions were: 5–27% B at 0.0–2.5 min, 27%–60% B at 2.5–7.0 min, 60%–75% B at 7.0–10.0 min, 75% B at 10.0–15.0 min, 75%–95% B at 15.0–18.0 min, 95%–100% B at 18.0–23.0 min, 100% B at 23.0–28.0 min and 5% B at 28.0–32.0 min.

A Q-TOF MS equipped with an electrospray ionization source (ESI) was used for positive and negative ionization scan modes (50–1,200 Da). The scanning mode was a full scan, and the time was 0.2 s. The detailed parameters of MS were: capillary voltage of 3,000 V (positive mode) and 2,200 V (negative mode), desolvation temperature at 350°C, sample cone voltage of 40 V, extraction cone voltage of 4 V, source temperature of 100°C, cone gas flow of 40 L/h and desolvation gas flow of 800 L/h (both positive and negative modes).

### 2.3 Rat Viral Pneumonia Models and Sampling

Sixty 6-8-week-old specific-pathogen free male Sprague Dawley (SD) rats (180–220 g) were purchased from Beijing Huafukang Bioscience Co. Ltd. (License number: SCXK (Beijing) 2020–0004) and fed at 12-h light-dark cycle with free access to water and food, at 21 ± 2°C with a relative humidity of 45 ± 10%. All rats were randomly divided into six groups (*n* = 10 per group): control group, model group, positive-drug group, QWJR low-dose (QWJR-L) group, QWJR middle-dose (QWJR-M) group and QWJR high-dose (QWJR-H) group. Among the three QWJR groups, the dosage ratio was 1:2:4, and the QWJR-M group was clinical practice. Hence, the dosage of QWJR-M group was based on the conversion of human to rat body surface area (1:6), which means the daily dose of QWJR for QWJR-M group rats should be 6 times that for human (Reagan-Shaw, 2008). According to the following formula:

$$\frac{185\text{g}\left(\text{c}\text{r}\text{u}\text{d}\text{e}\;\text{h}\text{e}\text{r}\text{b}\text{s}\right)/\text{d}\left(\text{h}\text{u}\text{m}\text{a}\text{n}\;\text{d}\text{o}\text{s}\text{a}\text{g}\text{e}\right)}{70\text{k}\text{g}\left(\text{h}\text{u}\text{m}\text{a}\text{n}\;\text{w}\text{e}\text{i}\text{g}\text{h}\text{t}\right)}\times 6.00\approx 15.86\;\text{g}/\text{k}\text{g}/\text{d}$$

the dosage for QWJR-L, QWJR-M and QWJR-H groups were 7.93, 15.86 and 31.72 g crude herb/kg/d. Considering the capacity of rat stomach, the extracted QWJR mixture was evaporated to 6.00 g(herbs)/mL (QWJR mixture), and the volume of concentrated QWJR mixture used was 1.32, 2.64 and 5.28 ml/kg/d for rats in QWJR-L, QWJR-M and QWJR-H groups respectively. The experiment was approved by the Ethics Committee of the Yunnan University of TCM.

**FIGURE 1:**
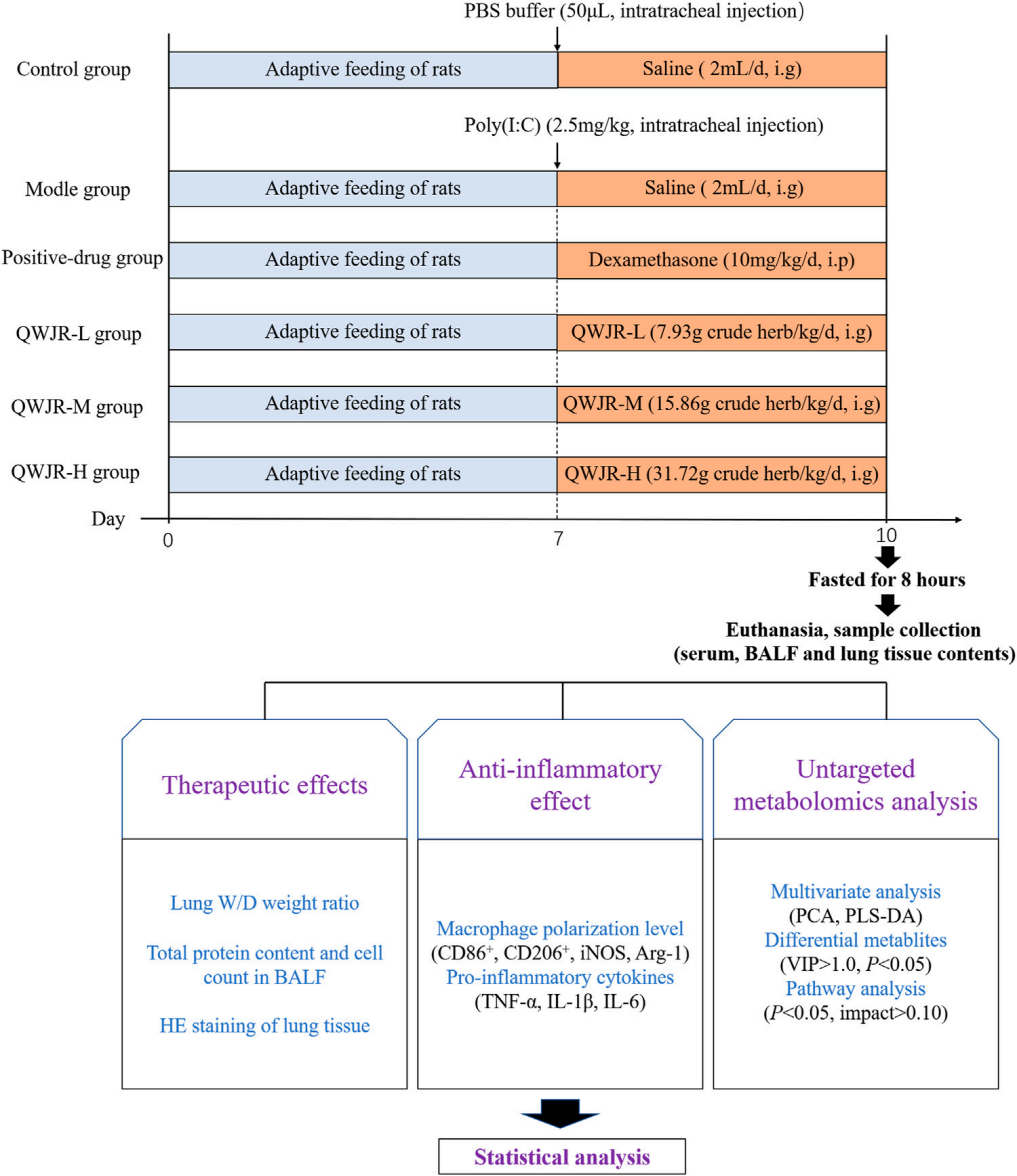
Overview of the experimental design for all groups.

After 1 week of acclimation, under anesthesia (pentobarbital sodium, 50 mg/kg), rats in the control group were intratracheally injected with 50 μL phosphate buffer saline (PBS), and other groups with 2.5 mg/kg poly (I:C) dissolved in 50 μL PBS, to establish the rat viral pneumonia model (Cui et al., 2020). After the intratracheal injection, control and model groups were given 2 ml saline/day via intragastric administration; the positive-drug group was given 10 mg/kg/day of dexamethasone via intraperitoneal injection (Ottolini et al., 2002); and the QWJR-L, QWJR-M and QWJR-H groups were given 1.32, 2.64 and 5.28 ml/kg of concentrated QWJR mixture (dissolved in 2 ml saline) via intragastric administration once a day, respectively, totally 3 days of intervention (Figure 1).

Serum, BALF and tissue samples were collected after intervention. For serum collection, rats were fasted for 8 h and anesthetized, and blood was collected via the abdominal aorta and centrifuged (3,000 rpm, 15 min, 4°C) to collect serum. Then the right main bronchus was ligated at the bifurcation after sacrificing. The BALF was obtained by injecting 2 ml of cold PBS into left bronchoalveolar using a syringe through the trachea three times. The collected BALF was centrifuged (2,000 rpm, 10 min, 4°C) to collect the supernatant and the precipitate. Finally, the right lung was extracted for wet/dry weight (W/D) ratio, histological and biochemical marker analysis.

### 2.4 Alveolar Capillary Barrier Tests

#### 2.4.1 W/D Ratio in Lung

The lower lobe of the right lung was placed on dry weighing paper to gain wet weight (W), and dry weight (D) was obtained by drying in a thermostat at 80°C for 48 h. The W/D ratio was calculated.

#### 2.4.2 Total Cell Count Test

The BALF precipitate was resuspended with 1 ml of PBS and mixed well using a pipette. Then, 10 μl cell suspension was added to the blood cell counting plate, and the total number of cells was counted under 40 × 10 magnification.

#### 2.4.3 Total Protein Concentration Test

Total protein concentration from the BALF supernatant was measured using BCA protein assay, according to the manufacturer’s instructions.

#### 2.4.4 Lung Tissue Pathology Staining

Tissue from the middle lobe of the right lung was fixed in 10% formalin solution, dehydrated, paraffin-embedded, and 5 µm sections were stained with hematoxylin and eosin (HE) for pathology analysis.

#### 2.4.5 Cytokines Expression Levels Tests

Cytokines (IL-1β, IL-6, and TNF-α) of the BALF supernatant were measured using ELISA kits, according to the manufacturer’s instructions.

#### 2.4.6 Macrophage Polarization Level Test

Macrophage polarization level of the BALF precipitate was measured using flow cytometry. Briefly, BALF was centrifuged and the precipitate cells were resuspended by PBS to obtain a cell concentration of 10^6^ cells/ml. Then the cell suspension was labeled with anti-rat CD86 and CD206 antibodies according to the manufacturer’s instructions. Finally, after washing with PBS twice, cells were suspended in 100 μl of PBS and run on a BD FACSCanto Flow Cytometer (BD Biosciences, Franklin Lakes, NJ, United States). The data was analyzed by FlowJo software (version 10, Tree Star Inc., Ashland) and calculated macrophage polarization level (the CD86^+^/CD206^+^ ratio).

#### 2.4.7 Macrophage Polarization-Related Factors Tests

0.1 g tissue of the upper lobe of the right lung was sonicated in 900 µL saline to obtain the lung tissue homogenate, and macrophage polarization-related factors (iNOS, Arg-1) in the supernatant were assayed by relative kits according to the manufacturer’s instructions.

### 2.5 Untargeted Metabolomics Analysis

#### 2.5.1 Preparation of Serum Samples

The serum sample (100 μl) was added to 400 μl of 80% methanol, vortexed and placed in an ice bath for 5 min, followed by centrifuging for 20 min (15,000 g, 4°C). Then the supernatant was diluted with ultrapure water to 53% methanol content and centrifuged again for 20 min (15,000 g, 4°C). Finally, the supernatant was collected as the testing sample. Equal amounts of all samples were mixed and used as quality control (QC) samples.

#### 2.5.2 Chromatography and Mass Spectrometry Conditions

A vanquish ultra-high-performance liquid chromatography (UHPLC) system (ThermoFisher, Germany) coupled with an Orbitrap Q ExactiveTM HF mass spectrometer (Thermo Fisher, Germany) was used for chromatography by Novogene Co., Ltd. (Beijing, China). Briefly, a Hypesil Goldcolumn (C18) chromatographic column (2.1 mm × 100 mm, 1.9 μm) with a mobile phase consisting of (A) 0.1% formic acid and (B) methanol was used; the gradient elution was 2% B, 1.5 min; 2–100% B, 12.0 min; 100% B, 14.0 min; 100-2% B, 14.1 min; 2% B, 17 min; the column temperature was set to 40°C, with a flow rate of 0.2 ml/min and an injection volume of 2 μl.

For Q Exactive™ HF mass spectrometry, the simultaneous detection in positive and negative ion mode was applied using the ESI. The ESI source settings were: spray voltage of 3.2 kV, sheath gas flow rate of 40arb, aux gas flow rate of 10arb, the capillary temp of 320°C, and polarity of positive and negative mode. The scan range was 100–1,500 m/z, and the mode was full MS-ddMS2.

#### 2.5.3 Data Processing and Metabolite Identification

The Compound Discoverer 3.1 (CD3.1, ThermoFisher) software was used to perform peak alignment, peak picking, and quantitation for each metabolite based on the raw data files generated by UHPLC-MS/MS. The main parameters were set as follows: retention time tolerance, 0.2 min; actual mass tolerance, 5 ppm; signal intensity tolerance, 30%; signal/noise ratio, 3; and minimum intensity, 100,000. After that, peak intensities were normalized to the total spectral intensity. The normalized data was used to predict the molecular formula based on additive ions, molecular ion peaks and fragment ions. And then, peaks were matched with the mzCloud (https://www.mzcloud.org/), mzVault and MassList database to obtain accurate qualitative and relative quantitative results. Statistical analyses were performed using the statistical software R (R version R-3.4.3), Python (Python 2.7.6 version) and CentOS (CentOS release 6.6). When data were not normally distributed, normal transformations were attempted using of area normalization method.

#### 2.5.4 Data Analysis

Resulted metabolites were annotated using the KEGG database (https://www.genome.jp/kegg/pathway.html), HMDB database (https://hmdb.ca/metabolites) and LIPIDMaps database (http://www.lipidmaps.org/). Principal components analysis (PCA) and partial least squares discriminant analysis (PLS-DA) were performed at metaX (a flexible and comprehensive software for processing metabolomics data). Univariate analysis (*t*-test) was applied to calculate the statistical significance (*p*-value). The metabolites with the variable importance in the projection (VIP) > 1 and *p* -value< 0.05 and fold change (FC) > 1.25 or FC < 0.8 were considered to be differential metabolites.

Pathway enrichment analysis was performed on differential metabolites based on MetaboAnalyst software (https://www.metaboanalyst.ca/) and Kyoto Encyclopedia of Genes and Genomes KEGG data (https://www.kegg.jp/).

#### 2.5.5 Statistics

All data were expressed as the mean ± standard deviation (mean ± SD) for independent experiments. One-way analysis of variance (one-way ANOVA) was used for comparison among multiple groups using SPSS software (version 20.0), and a *p* < 0.05 was taken as statistically significant. The GraphPad Prism 5 software was used for curve fitting.

## 3 Results

### 3.1 Identification of Main Compounds in QWJR by UPLC-MS Analysis

Chlorogenic acid, forsythoside A, liquiritin, saikosaponin A, baicalin, arginine, magnolol, protocatechuic acid, 4-hydroxyacetophenone, aristolochic acid A and artemisinin were used as the reference standards to validate the main compounds in QWJR. The detailed information on these compounds were shown in Supplementary Figure S2. The typical based peak intensity chromatograms of QWJR and these reference standards were shown in Supplementary Figure S3. The characteristic fragment ions of these compounds were shown in Supplementary Table S1. Chlorogenic acid in *Valeriana jatamansi* Jones ex Roxb., forsythoside A in *Forsythia suspensa* (Thunb.) Vahl, liquiritin in *Glycyrrhiza uralensis* Fisch. ex DC., saikoponin A in *Bupleurum candollei* Wall. ex DC., baicalin in *Scutellaria amoena* C.H.Wright, arginine in *Pinellia ternata* (Thunb.) Makino, magnolol in *Magnolia officinalis* Rehder & E.H.Wilson, protocatechuic acid in *Hedychium spicatum* Sm., 4-hydroxyacetophenone in *Vincetoxicum atratum* (Bunge) C.Morren & Decne., aristolochic acid A in *Magnolia officinalis* Rehder & E.H.Wilson and artemisinin in *Artemisia capillaris* Thunb*.* were identified as the preeminent compounds of QWJR.

### 3.2 Therapeutic Effects of QWJR on Poly (I:C)-Induced Viral Pneumonia Rats

**FIGURE 2:**
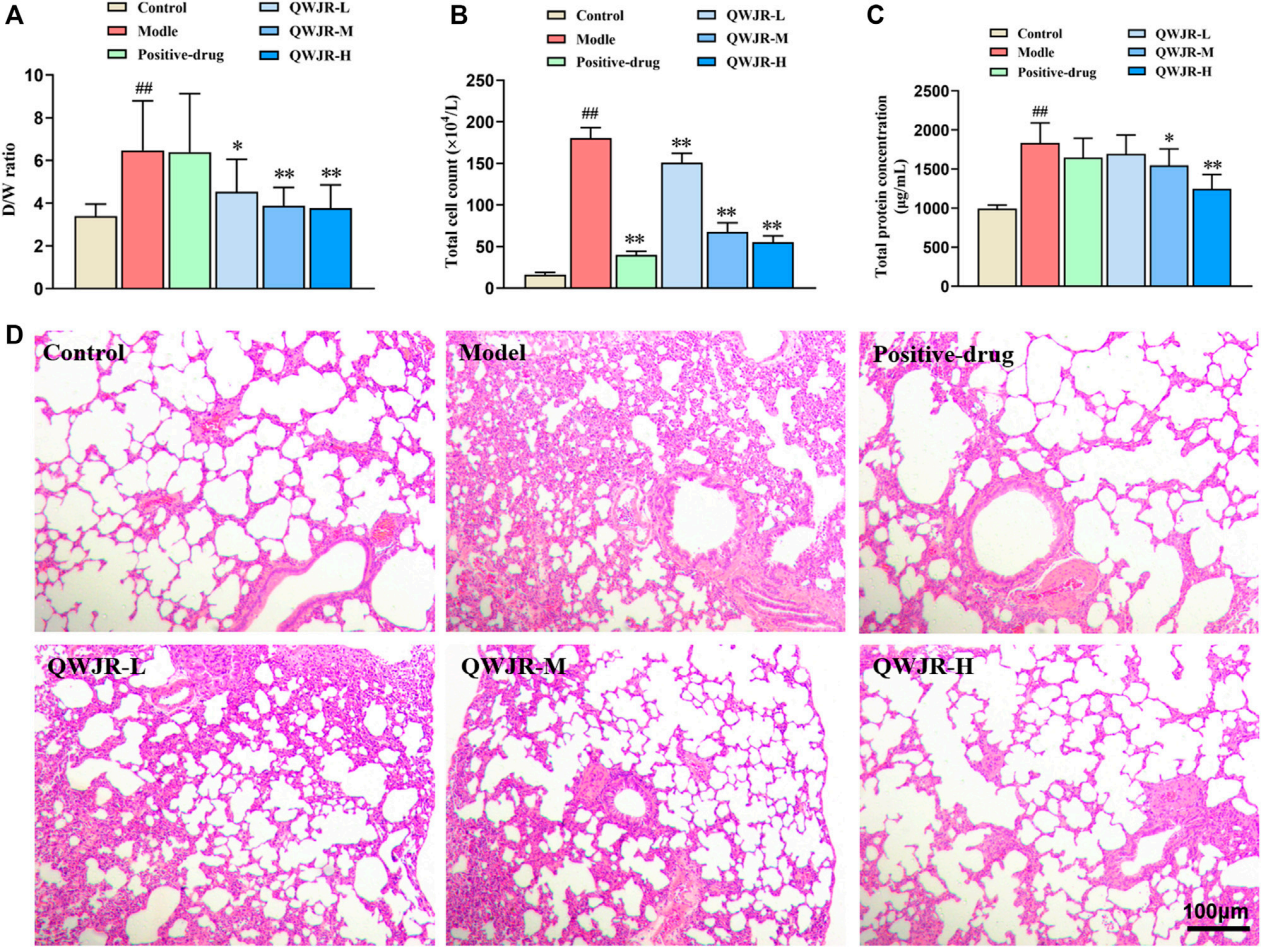
Therapeutic effects of QWJR on poly (I:C)-induced viral pneumonia rats. **(A)** The W/D ratio was decreased after QWJR treatment. **(B)** The total cell count was decreased after positive-drug and QWJR treatment. **(C)** The total protein concentration was decreased after QWJR treatment. **(D)** After positive-drug and QWJR intervention, the histopathological changes in the rat lungs were reduced (200×). Control, model, positive-drug, QWJR-L, QWJR-M, and QWJR-H (*n* = 10 per group) groups. Data are presented as the mean ± SD. ^##^: *p* < 0.01 as compared to the control group; ^*^: *p* < 0.05 as compared to the model group; ^**^: *p* < 0.01 as compared to the model group.

After intratracheal injection of poly(I:C) in rats, the W/D ratio, total cell count and total protein concentration were significantly increased in the model group compared with those in the control group (*p* < 0.01, respectively) (Figure 2). While after QWJR administration, the W/D ratio was decreased in the QWJR-L (*p* < 0.05), QWJR-M (*p* < 0.01) and QWJR-H (*p* < 0.01) groups compared with the model group (Figure 2A). The total cell count was decreased in the positive-drug, QWJR-L, QWJR-M and QWJR-H groups compared with the model group (*p* < 0.01, respectively) (Figure 2B), and the total protein concentration was decreased in the QWJR-M (*p* < 0.05) and QWJR-H (*p* < 0.01) groups compared with the model group (Figure 2C).

HE staining of the lung tissues showed that the bronchial and alveolar structures of control group rats were intact, and no significant pulmonary interstitial hyperemia or inflammatory cell infiltration were observed. In the model group, the alveolar wall was widened and edematous in most areas, and the alveolar was even collapsed, with a great extent of inflammatory cell infiltration and erythrocyte exudation. After dexamethasone and QWJR intervention, the histopathological changes were found to reduce in the positive-drug, QWJR-L, QWJR-M, and QWJR-H groups (Figure 2D).

Role of QWJR on the inflammatory response of poly(I:C)-induced viral pneumonia rats.

**FIGURE 3:**
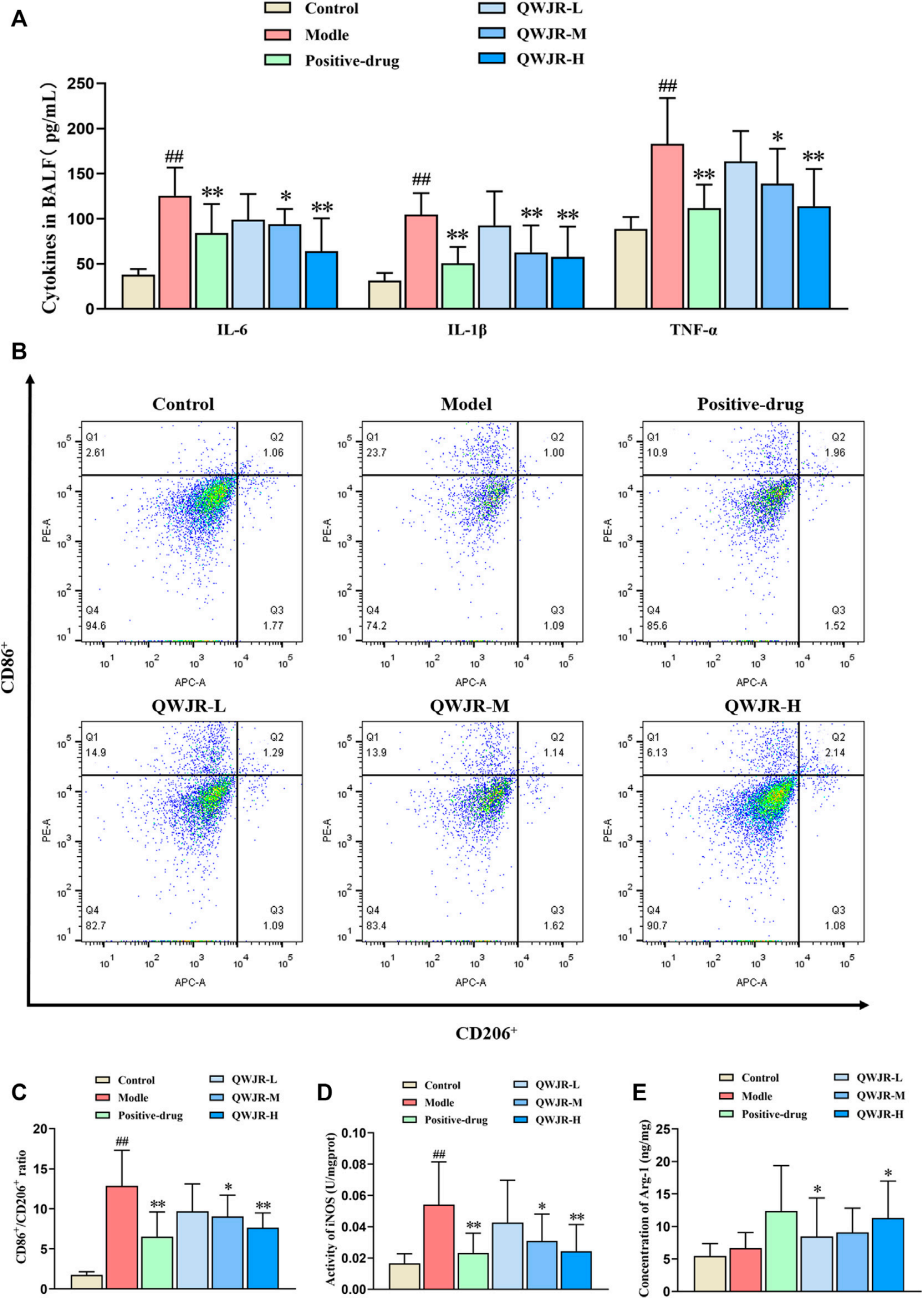
Role of QWJR on the inflammatory response of poly(I:C)-induced viral pneumonia rats. **(A)** The levels of pro-inflammatory cytokines IL-6, IL-1β and TNF-α were decreased after positive-drug and QWJR treatment. **(B,C)** The ratio of CD86^+^/CD206^+^ was decreased after positive-drug and QWJR treatment. **(D)** The activity of iNOS was decreased after positive-drug and QWJR treatment. **(E)** The expression level of Arg-1 was increased after positive-drug and QWJR treatment. Control, model, positive-drug, QWJR-L, QWJR-M, and QWJR-H (*n* = 10 per group) groups. Data are presented as the mean ± SD. ^##^: *p* < 0.01 as compared to the control group; ^*^: *p* < 0.05 as compared to the model group; ^**^: *p* < 0.01 as compared to the model group.

For anti-inflammatory effects, expression levels of pro-inflammatory cytokines IL-6, IL-1β and TNF-α were measured by ELISA. As shown in Figure 3A, expression of IL-6, IL-1β and TNF-α in the BALF significantly increased in the model group compared with control group (*p* < 0.01, respectively), and decreased in the positive-drug group (*p* < 0.01 respectively), QWJR-M (*p* < 0.05, *p* < 0.01 and *p* < 0.05, respectively) and QWJR-H (*p* < 0.01, respectively) group.

Macrophage polarization level was represented by the ratio of CD86^+^/CD206^+^ cells. As shown in Figures 3B,C, the ratio of CD86^+^/CD206^+^ was significantly higher in model group than control group (*p* < 0.01). However, the ratio was decreased in the positive-drug group (*p* < 0.01), QWJR-M (*p* < 0.05) and QWJR-H (*p* < 0.01) groups compared with the model group (Figures 3B,C). M1-type macrophage marker (iNOS) and M2-type macrophage marker (Arg-1) expression levels showed that iNOS was significantly increased (*p* < 0.01) in model group than control group, and significantly decreased in the positive-drug group (*p* < 0.01), QWJR-M (*p* < 0.05) and QWJR-H (*p* < 0.01) (Figure 3D); whereas Arg-1 expression level was only significantly increased in the positive-drug group (*p* < 0.05) and QWJR-H group (*p* < 0.05) (Figure 3E).

### 3.3 Effect of QWJR on Serum Metabolite Levels in Poly(I:C)-Induced Viral Pneumonia Rats

**FIGURE 4:**
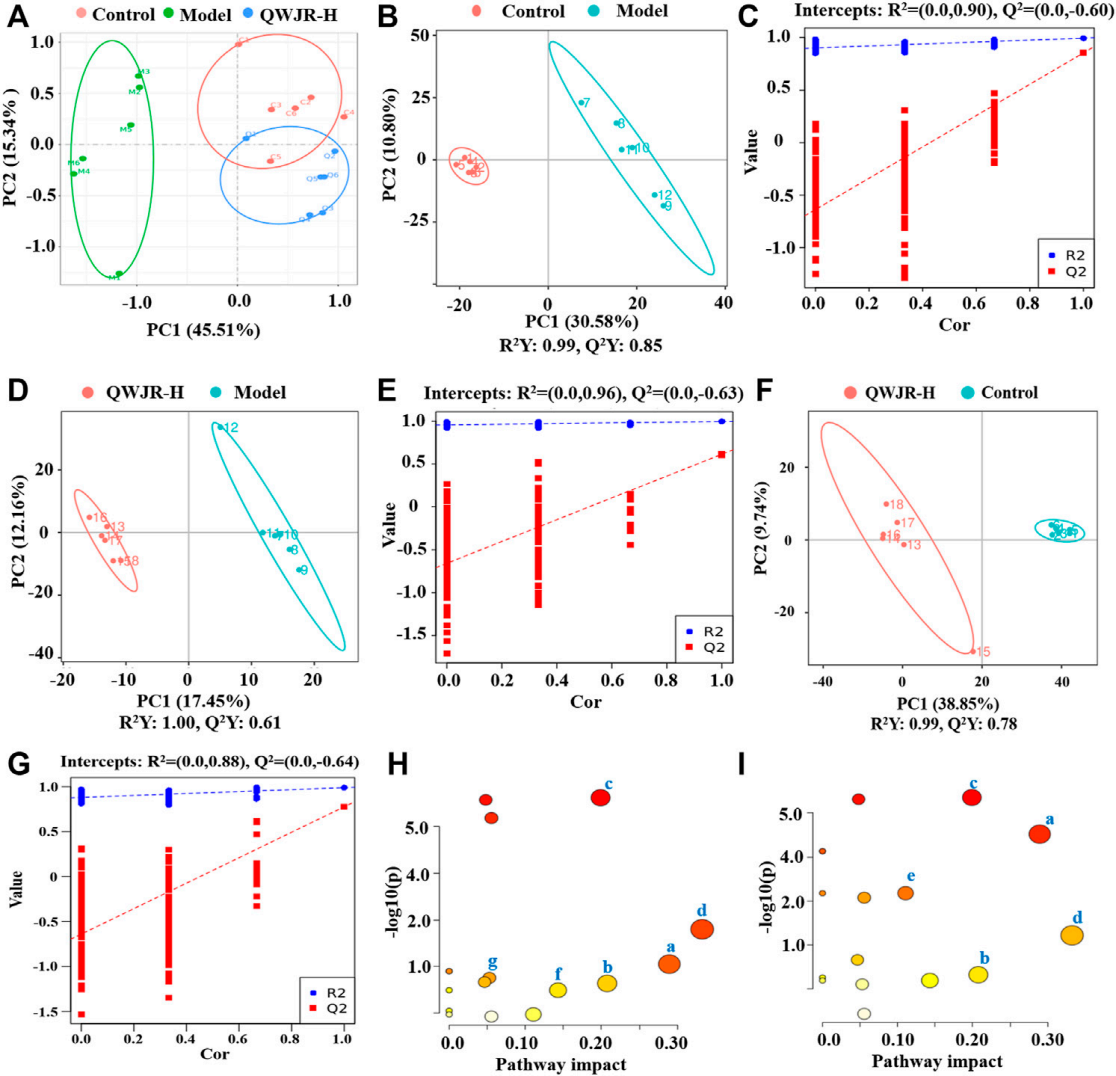
Effect of QWJR on serum metabolite levels in poly(I:C)-induced viral pneumonia rats. **(A)** Scores plots of PCA between the control and model groups and the model and QWJR-H groups. **(B,C)** Scores plots of PLS-DA between the control and model groups and the corresponding coefficient of loading plots. **(D,E)** Scores plots of PLS-DA between the model and QWJR-H groups and the corresponding coefficient of loading plots. **(F,G)** Scores plots of PLS-DA between the control and QWJR-H groups and the corresponding coefficient of loading plots. **(H,I)** Summary of pathway analysis of serum samples between control and model groups and between model and QWJR-H groups. a: Arginine metabolism; b: Arachidonic acid metabolism; c: Citrate cycle; d: Nicotinate and nicotinamide metabolism; e: Arginine and proline metabolism; f: Tryptophan metabolism; g: Porphyrin and chlorophyll metabolism. Control, model and QWJR-H (*n* = 6 per group) groups.

An unsupervised and comprehensive view of PCA was constructed to explore the distribution and tendencies of control, model, and QWJR-H groups. A good separation trend among the three groups was shown in the PCA plots (Figure 4A). Likewise, a supervised PLS-DA analysis was used to sharpen the separation among groups and enhances the recognition of variables that contribute to categorical. As shown in Figures 4B,D,F, all the models exhibited excellent explanatory and predictive with R^2^Y > Q^2^Y. In addition, the evaluation parameters (*R* ^2^, Q^2^) of PLS-DA model were obtained using seven-round cross-validation and 200 repetitions of response permutation testing. The model group had *R* ^2^ = 0.90 and Q^2^ = −0.60 compared to the control group (Figure 4C), the QWJR-H group had *R* ^2^ = 0.96 and Q^2^ = −0.63 compared to the model group (Figure 4E), and the QWJR-H group had *R* ^2^ = 0.88 and Q^2^ = −0.64 compared to the control group (Figure 4G). The 200-times permutation test showed that all the established PLS-DA models were credible and not overfitting, as the *R* ^2^ data is larger than Q^2^ data and the intercept between Q^2^ regression line and *Y*-axis is less than 0. The differences between experimental groups were greater than within groups. Suggesting that the serum metabolic profiles after poly(I:C) and QWJR treatment had changed dramatically.

Simultaneously, the screening of differential metabolites mainly refers to VIP, FC and *p*-value of metabolites. VIP refers to the variable importance in the projection of the first principal component of PLS-DA model, and the VIP value represents the contribution of metabolites to grouping. FC refers to the fold change. *p*-value is calculated by *t*-test and represents the significance level of difference. FC > 1.25 or FC < 0.8, VIP >1 and *p* < 0.05 were used as criteria. According to the above criteria, variable ions were identified. A series of matched results were then obtained after searching online databases such as mzCloud, mzVault, and MassList.

**FIGURE 5:**
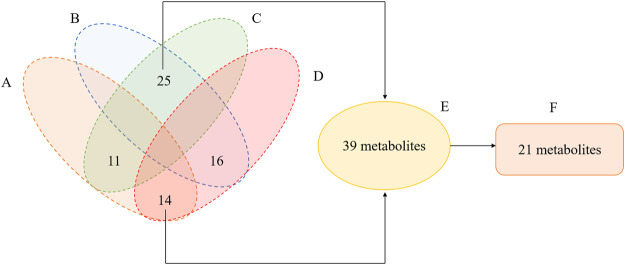
Venn diagram illustrated the overlapping and unique differential metabolites among the comparison groups. **(A)** metabolites showed elevated levels in the M vs. C group. **(B)** metabolites showed decreased levels in M vs. C group. **(C)** metabolites showed elevated levels in the Q vs. M group. **(D)** metabolites showed decreased levels in Q vs. M group. **(E)** metabolites showed opposite variation trends between M vs. C and Q vs. M. **(F)** metabolites had a close expression level in Q vs. C group.

A total of 809 metabolites were detected in the serum of all the three experimental groups. Differential metabolites were then screened according to the following criteria: FC > 1.25 or FC < 0.8, *p* < 0.05 and VIP >1.0. The number of differential metabolites were 176, 167 and 105, respectively, in the comparison groups of M vs. C, Q vs. M and Q vs. C. 66 metabolites were found to vary in both the M vs. C and Q vs. M groups (Supplementary Table S2), of which 39 metabolites showed opposite variation trends. Among the 39 metabolites, 21 metabolites had a comparable expression level in Q vs. C group, which could be the pivotal differential metabolites associated with the therapeutic effect of QWJR (Table 2). The screening method was shown in Figure 5 (Yang et al., 2021).

**TABLE 2:** The differential metabolites associated with the therapeutic effect of QWJR in serum.

No.	Formula	RTw [min]	m/z	Metabolites	VIP			FC			Trend			Pathway
					M vs. C	Q vs. M	Q vs. C	M vs. C	Q vs. M	Q vs. C	M vs. C	Q vs. M	Q vs. C	
1	C_6_H_13_N_3_O_3_	1.38	174.09	Citrulline	1.58	1.21	0.88	0.59	1.67	1.01	↓^##^	↑^*^	—	a
2	C_20_H_32_O_3_	8.56	343.22	16(R)-HETE	1.40	1.90	0.48	0.47	2.56	1.00	↓^#^	↑^**^	—	b
3	C_18_H_30_O_3_	8.24	317.21	13-OxoODE	1.86	1.23	0.10	0.44	1.62	1.00	↓^##^	↑^*^	—	
4	C_5_H_6_O_5_	1.55	145.01	alpha-Ketoglutaric acid	1.62	1.96	0.37	0.39	1.78	1.00	↓^##^	↑^*^	—	c+a
5	C_12_H_18_O_3_	6.43	209.12	Jasmonic acid	1.37	1.34	0.27	0.41	1.46	0.99	↓^##^	↑^*^	—	
6	C_22_H_32_O_2_	10.09	327.23	Docosahexaenoic acid	1.44	1.05	0.72	1.54	0.72	1.01	↑^#^	↓^**^	—	
7	C_20_H_32_O_6_	7.24	367.21	Prostaglandin G2	1.59	2.21	0.73	1.88	0.22	0.99	↑^#^	↓^**^	—	b
8	C_33_H_46_N_4_O_6_	5.78	595.35	Stercobilin	1.08	2.05	0.34	0.16	3.73	1.00	↓^#^	↑^**^	—	g
9	C_28_H_44_O	8.00	397.34	Ergocalciferol	1.86	1.18	0.66	0.22	1.83	1.01	↓^##^	↑^**^	—	
10	C_7_H_8_N_2_O	1.39	137.07	1-Methylnicotinamide	1.44	1.27	0.86	2.60	0.37	0.98	↑^#^	↓^*^	—	d
11	C_18_H_16_O_8_	5.11	356.13	Rosmarinic acid	1.61	1.25	0.74	1.60	0.76	1.01	↑^#^	↓^**^	—	
12	C_5_H_11_NO_2_	1.39	118.09	Betaine	1.06	1.53	0.26	0.65	1.54	1.04	↓^#^	↑^**^	—	
13	C_20_H_32_O_5_	6.59	333.21	Prostaglandin D2	1.37	1.42	0.15	0.60	3.16	1.01	↓^#^	↑^**^	—	b
14	C_33_H_36_N_4_O_6_	7.55	585.27	Bilirubin	1.66	1.24	0.11	2.81	0.27	1.00	↑^##^	↓^*^	—	g
15	C_18_H_22_O_2_	6.98	253.16	Estrone	1.03	1.28	0.68	2.11	0.20	1.00	↑^##^	↓^*^	—	
16	C_20_H_32_O_5_	8.18	333.21	Prostaglandin H2	1.43	1.73	0.06	0.49	6.82	1.01	↓^#^	↑^**^	—	b
17	C_6_H_6_O_6_	1.45	173.01	cis-Aconitic acid	1.29	1.68	0.18	0.25	3.55	1.02	↓^##^	↑^**^	—	c
18	C_6_H_8_O_7_	1.55	191.02	Citric acid	1.65	1.17	0.05	2.13	0.76	0.98	↑^##^	↓^*^	—	c
19	C_6_H_6_N_2_O	2.01	123.06	Nicotinamide	1.47	1.34	0.39	2.11	0.21	1.00	↑^#^	↓^*^	—	d
20	C_11_H_12_N_2_O_2_	6.79	203.08	L-Tryptophan	1.73	1.08	0.16	0.41	2.72	1.01	↓^##^	↑^*^	—	f
21	C_5_H_12_N_2_O_2_	1.19	133.10	L-Ornithine	1.60	1.44	0.24	0.33	1.45	1.00	↓^##^	↑**	—	e+a

Control, model and QWJR-H (*n* = 6 per group) groups.

^#^: *p* < 0.05 as compared to the control group; ^##^: *p* < 0.01 as compared to the control group; ^*^: *p* < 0.05 as compared to the model group; ^**^: *p* < 0.01 as compared to the model group; ↑: content increased (FC > 1.25); ↓: content decreased (FC < 0.8); —: content doesn’t change much (FC ≈ 1); vs, versus; C, control group; M, model group; Q, QWJR-H group.

a: Arginine metabolism; b: Arachidonic acid metabolism; c: Citrate cycle; d: Nicotinate and nicotinamide metabolism; e: Arginine and proline metabolism; f: Tryptophan metabolism; g: Porphyrin and chlorophyll metabolism.

The metabolic pathways of the differential metabolites were analyzed by the MetaboAnalyst software (*p* < 0.05 and impact value >0.10). The results revealed that metabolic pathways altered in the model group, compared to the control group, mainly arginine metabolism, AA metabolism, TCA cycle, nicotinate and nicotinamide metabolism, and arginine and proline metabolism (Figure 4H). Compared to the model group, the metabolic pathways affected by QWJR mainly included arginine metabolism, AA metabolism, TCA cycle, nicotinate and nicotinamide metabolism, tryptophan metabolism, and porphyrin and chlorophyll metabolism (Figure 4I). Based on the above metabolic pathway analysis results, the common metabolic pathways between the control and model groups and between the model and QWJR-H groups were arginine metabolism, AA metabolism, TCA cycle, and nicotinate and nicotinamide metabolism.

## 4 Discussion

In this study, we successfully generated a rat model of viral pneumonia via intratracheal injection of poly(I:C). Intervention with QWJR effectively ameliorated damage to alveolar-capillary barrier, as the lung W/D ratio, total cell count and total protein concentration of BALF were decreased. The histopathological damage in lung was improved, and the most significant therapeutic effect was observed in the high-dose group. Our results showed that a high dose of QWJR had a similar function to that of dexamethasone, which is a widely used anti-inflammatory drug in clinic (Cui et al., 2020), suggesting QWJR could be an alternative to dexamethasone for the treatment of viral pneumonia.

We chose poly(I:C) to simulate ribonucleic acid (RNA) viral pneumonia in this study based on its structural resemblance to the RNA of many viruses (Fortier et al., 2004; Cunningham et al., 2007). Apart from alveolar-capillary barrier integrity, the inflammatory response has an important role in the pathogenesis of viral pneumonia (Szeto, 2019; Bhaskar et al., 2020). The inflammatory cytokines analysis of this study demonstrated the upregulation of TNF-α, IL-1β, and IL-6 in viral pneumonia rats, which are thought to amplify the inflammatory response and aggravate lung injury through intercellular communication and signal transduction (Rabaan et al., 2021), could be reversed by QWJR administration. As a pro-inflammatory cytokine, TNF-α is mainly produced by activated macrophages, and an increased level reflects the severity of lung injury in patients (Roth et al., 2013). IL-1β regulates innate immunity, which has a wide range of biological activities and can induce the regulation of discharge and release of other inflammatory mediators in various cells (Dinarello, 2018). IL-6 is the most potent endogenous inflammatory cytokine that triggers the systemic inflammatory response, the expression level of IL-6 could reflect the severity of inflammation and disease in patients (De Gonzalo-Calvo et al., 2010).

Macrophages play an important role in the inflammatory response to viral infection (Arango Duque et al., 2014). They have two distinct statuses by polarization into classically activated macrophages (M1) and alternatively activated macrophages (M2) (Biswas et al., 2012; Sica and Mantovani, 2012). M1-type macrophages are induced by Th1 cytokines and are characterized by the discharge of pro-inflammatory factors, such as TNF-α, IL-1β, IL-12, and iNOS (Murray et al., 2014; Kim et al., 2018). M2-type macrophages are induced by Th2 cytokines, which suppress the inflammatory response by discharging immunomodulatory factors, such as Arg-1, interleukin-10 and transforming growth factor-β (Yang et al., 2018; Cheng et al., 2019). In the present study, the flow cytometry results revealed that QWJR diminished the ratio of CD86^+^/CD206^+^macrophages, decreased iNOS activity and increased Arg-1 expression level, indicating that QWJR could alleviate the inflammatory response of viral pneumonia by inhibiting the polarization of M1-type macrophages and promoting the polarization of M2-type macrophages.

We tried to clarify the complex pathophysiological processes of QWJR in treating viral pneumonia from the perspective of metabolomics analysis. A total of 21 differential metabolites were identified through untargeted metabolomics of serum, and further analysis indicated that the arginine metabolism, AA metabolism, TCA cycle and nicotinate and nicotinamide pathways might be involved in the treatment of poly (I:C)-induced viral pneumonia rats with QWJR.

### 4.1 Arginine Metabolism

Arginine and its metabolites are closely associated with inflammatory response and may inhibit the discharge of pro-inflammatory factors (Xiao et al., 2021). In this study, citrulline and L-ornithine, as the metabolites of arginine (Tsuei et al., 2005), were significantly reduced in poly(I:C)-induced viral pneumonia rats and elevated by QWJR treatment (Table 2). Citrulline could inhibit the release of inflammatory cytokines such as TNF-α, IL-6, and IL-1β (Cai et al., 2016) and scavenge hydroxyl radicals produced by severe stress, ischemia and hypoxia (Moinard and Cynober, 2007). L-ornithine is the precursor of polyamines and proline, which promote TCA cycle and oxidative phosphorylation and inhibit nitric oxide (NO) mediated inflammatory response and exert anti-inflammatory effects (Kieler et al., 2021).

### 4.2 AA Metabolism

AA and its metabolites are strongly associated with the development and regression of inflammation (Calder, 2009; Yao et al., 2009; Legler et al., 2010; Bosma-den Boer et al., 2012). Under catalyzation of cyclooxygenase, AA can generate unstable PGG2, and rapidly convert to PGH2, which is finally converted to biologically active PGD2 by various enzymes (Hamberg et al., 1975; Cha et al., 2006). Our results showed that 16R-HETE, PGH2, and PGD2 were decreased, and PGG2 was increased in poly(I:C)-induced viral pneumonia rats. 16R-HETE is a potent adhesion inhibitor of human polymorphonuclear leukocytes, and it selectively inhibits the adhesion and aggregation of neutrophils (Bednar et al., 2000). PGD2 is an eicosanoid with inflammatory modulating properties (Hirata et al., 1994; Vijay et al., 2017). When inflammation occurs, PGD2 could bind to the prostaglandin receptor 1 (DP1) of macrophages, block the polarized janus kinase 2/signal transducer and activator of transcription 1 (JAK2/STAT1) signaling pathway of M1-type macrophage, thereby promote the polarization of M2-type macrophages (Kong et al., 2016). After QWJR intervention, 16R-HETE, PGH2 and PGD2 were elevated, while PGG2 decreased. Thus, QWJR may take effect by regulating the activity of key enzymes in AA metabolism in this study.

### 4.3 TCA Cycle

The TCA cycle provides an energy source for organisms, and the metabolites accumulated from the TCA cycle can affect immune cells’ function (Martínez-Reyes and Chandel, 2020). A study found that in patients with COVID-19, TCA cycle was one of the most affected metabolic pathways (Jia et al., 2021). In the present study, viral pneumonia rats had elevated levels of citric acid and decreased levels of cis-aconitic acid compared with normal rats. However, after QWJR intervention, citric acid decreased and cis-aconitic acid increased, returning to normal. Citric acid is not only the primary source of adenosine triphosphate (ATP) but also an inflammatory signal whose accumulation may help generate pro-inflammatory mediators, such as NO and reactive oxygen species (ROS) (Infantino et al., 2014; Fan et al., 2021). In viral pneumonia, the TCA cycle in M1-type macrophages is blocked, leading to citric acid accumulation and succinate. As the material basis for the inflammatory function of M1-type macrophages (Corcoran and O'Neill, 2016), citric acid accumulation could further accelerate glycolytic metabolism and promote the inflammatory response (Mills et al., 2016), leading to the exacerbation of lung damage. The cis-aconitic acid is an intermediate product of citric acid isomerization, which is further decarboxylated to itaconic acid and involved in the anti-inflammatory responses of macrophages (Chouchani et al., 2014; Yu et al., 2019), by inhibiting the expression of inflammatory cytokines like IL-β, IL-6 and TNF-α, and exerts anti-inflammatory effects (Li Y. et al., 2020).

### 4.4 Nicotinate and Nicotinamide Metabolism

Nicotinate and nicotinamide metabolism is closely related to energy metabolism, oxidative stress response, etc. (Maiese et al., 2009). Nicotinamide is a derivative of nicotinic acid and has a wide range of cytoprotective effects (Maiese et al., 2009). It helps restore ATP levels by repairing mitochondrial function while inhibiting activation of excessive poly adenosine diphosphate ribose polymerase. Then the cell necrosis rate could be reduced and the overexpression of nuclear factor kappa-B could be hindered, so that the production of pro-inflammatory cytokines and other inflammatory mediators could be reduced (Exline and Crouser, 2008), and the development of inflammatory response could also be prevented (Fang et al., 2016; Pissios, 2017). Furthermore, nicotinamide was proved to inhibit the expression of iNOS and release free radicals and pro-inflammatory cytokines (Su et al., 2007). Studies have found that nicotinamide could reduce bleomycin-induced acute lung injury (Nagai et al., 1994) and mitigate the inflammatory response of ventilator-induced lung injury (Zhang and Liu, 2020). In this study, we found nicotinamide decreased and the primary metabolite of nicotinamide 1-methylnicotinamide increased in the viral pneumonia rats, while inversely after QWJR intervention. Indicating that QWJR may help alleviate acute lung injury induced by viral infection through restoring nicotinamide, which is effective in regulating host immune response and preventing cytokine storm (Gharote, 2020).

## 5 Conclusion

In conclusion, QWJR can be used as an alternative therapy for viral pneumonia and alleviate acute lung injury caused by the virus. The mechanisms are associated with inhibiting inflammatory response, modulating arginine, AA, TCA cycle, and nicotinate and nicotinamide metabolism processes.

## Data Availability

The original contributions presented in the study are included in the article/Supplementary Material, further inquiries can be directed to the corresponding authors.
