# Supplementary material for: Qing-Wen-Jie-Re Mixture Ameliorates Poly (I:C)-Induced Viral Pneumonia Through Regulating the Inflammatory Response and Serum Metabolism
Source: Front Pharmacol. 2022 Jun 15;13:891851. doi: 10.3389/fphar.2022.891851 (PMC9240632; doi:10.3389/fphar.2022.891851)
Supplement: Supplementary file 1 [file Table1.DOCX]

Supplementary Material


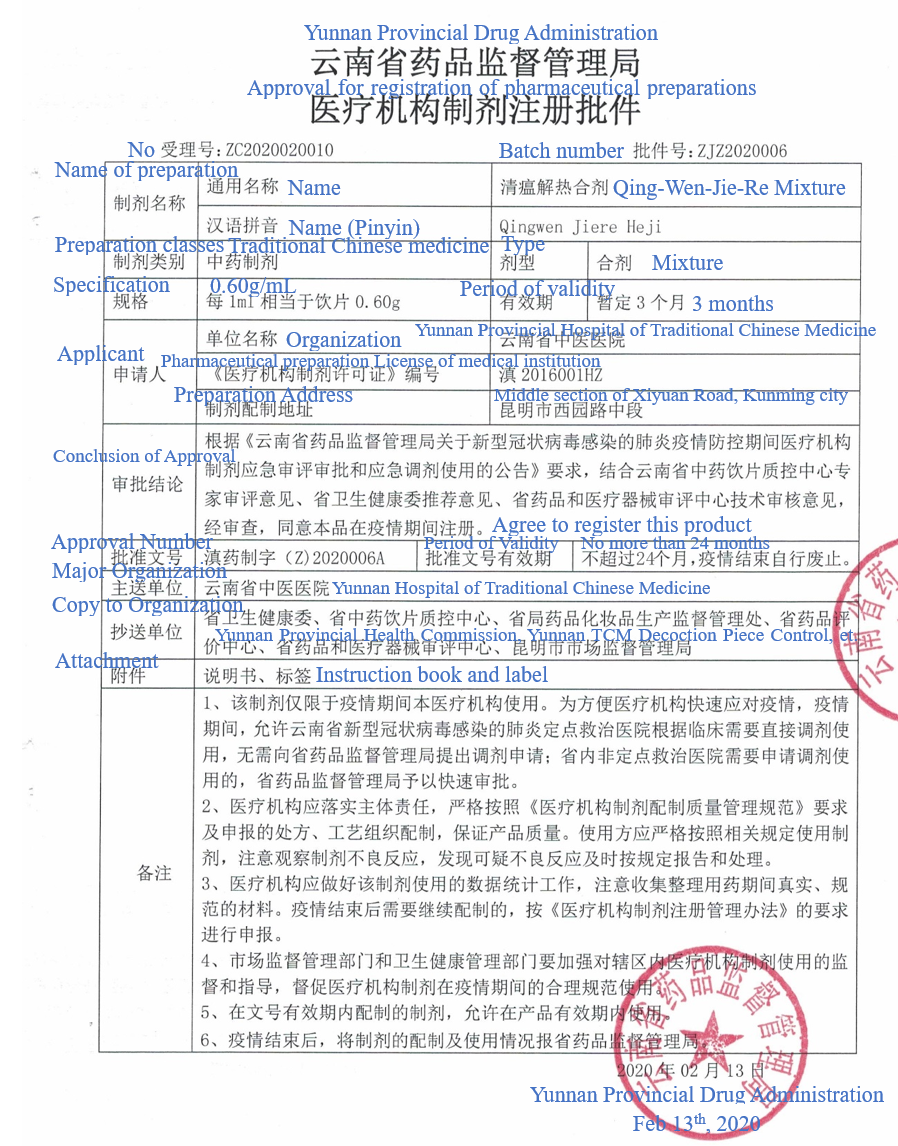


**FIGURE S1:** The production licence of QWJR

s

**
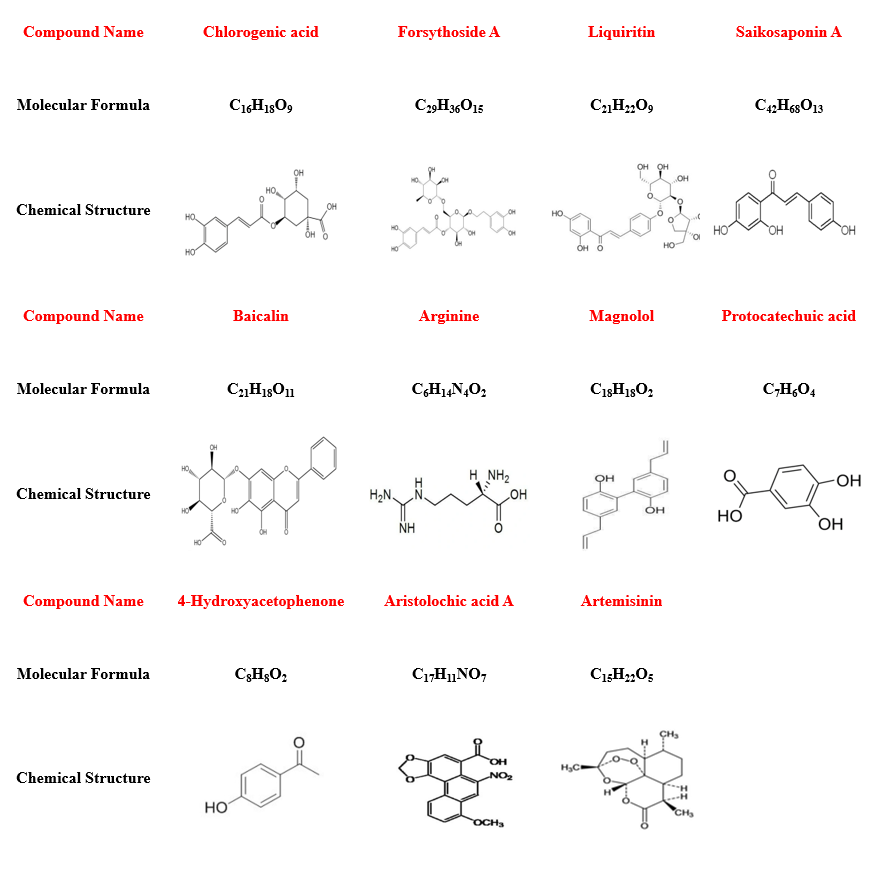
**

**FIGURE S2:** The molecular formulas and chemical structures of reference

**A**


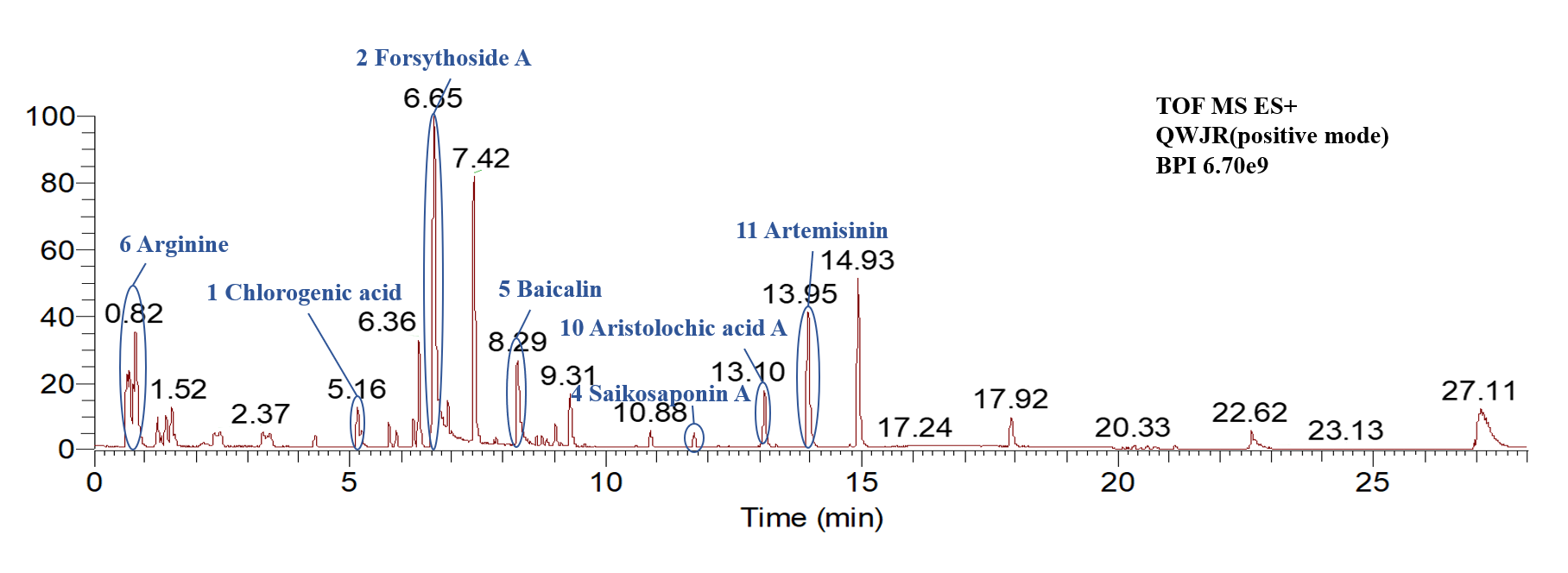


**B**


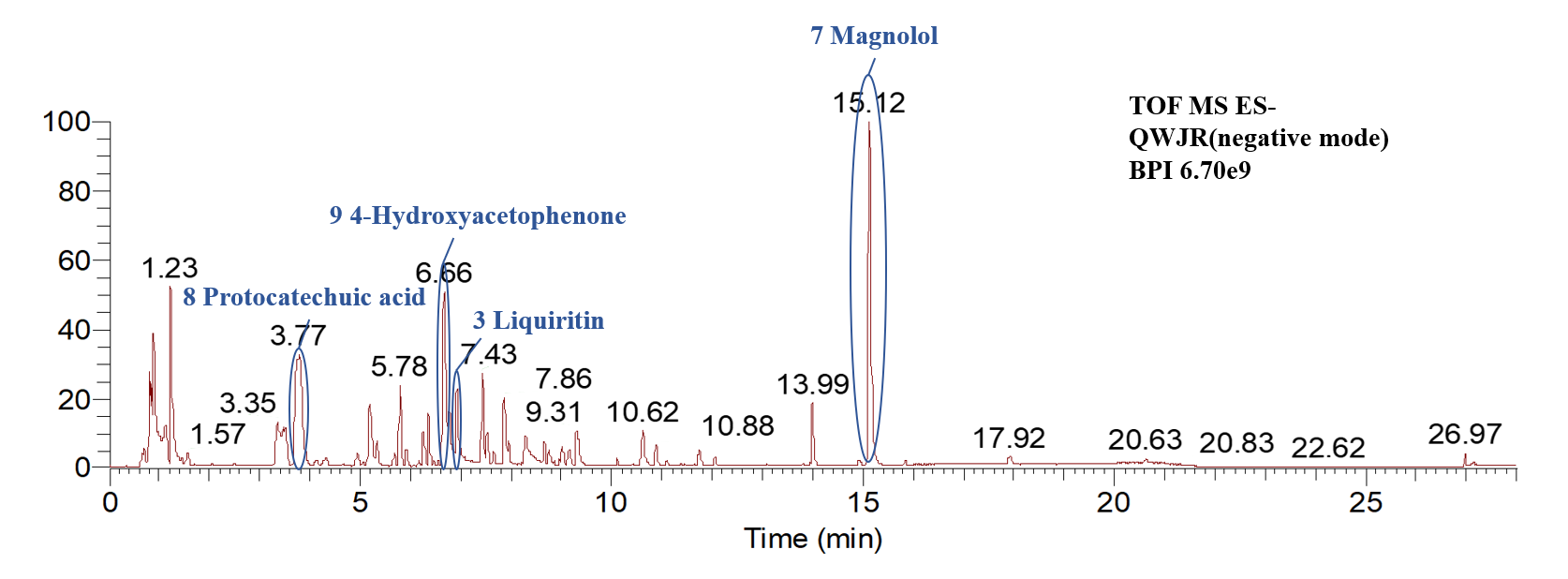


**C**

**
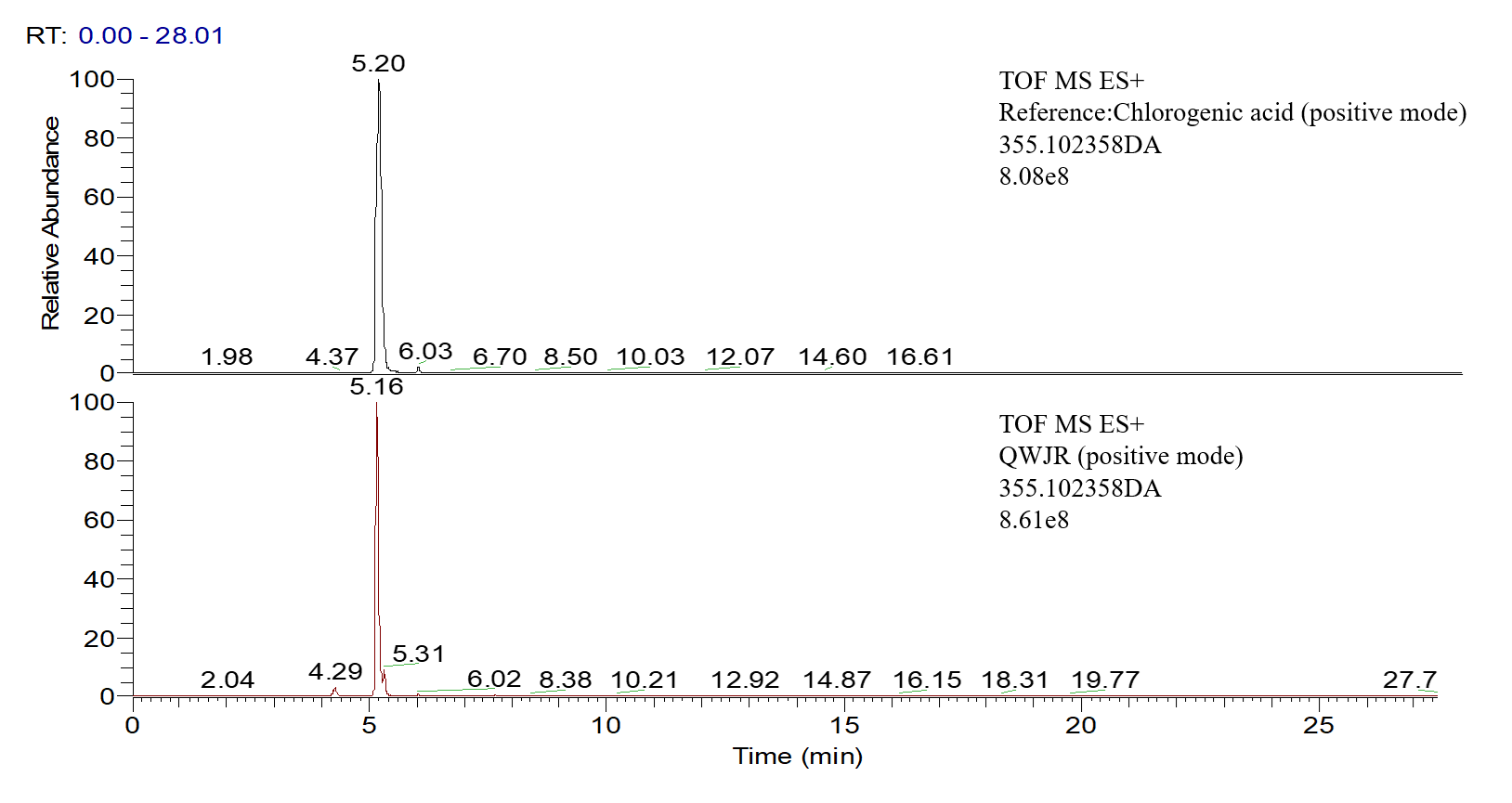
**

**D**

**
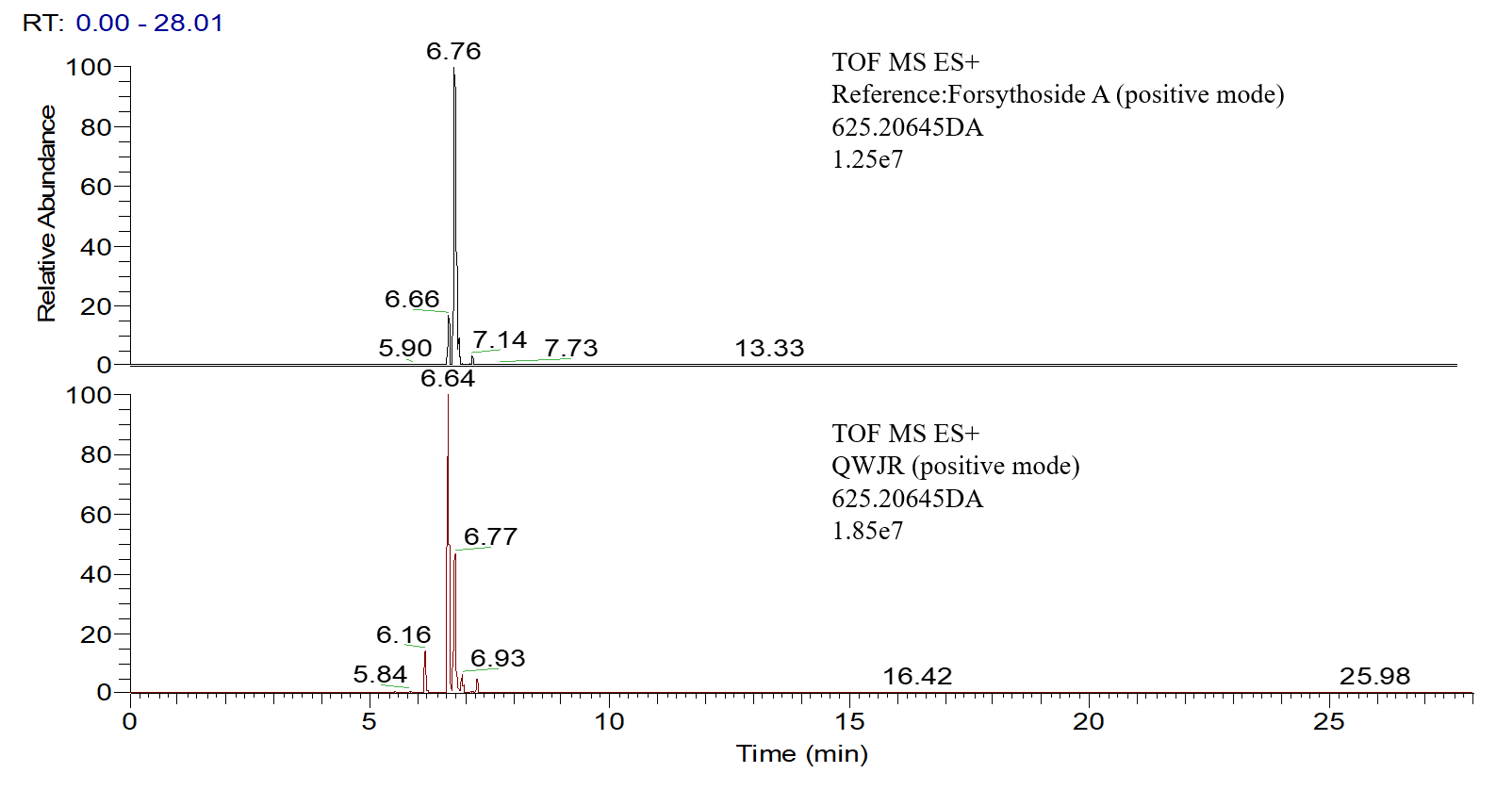
**

**E**

**
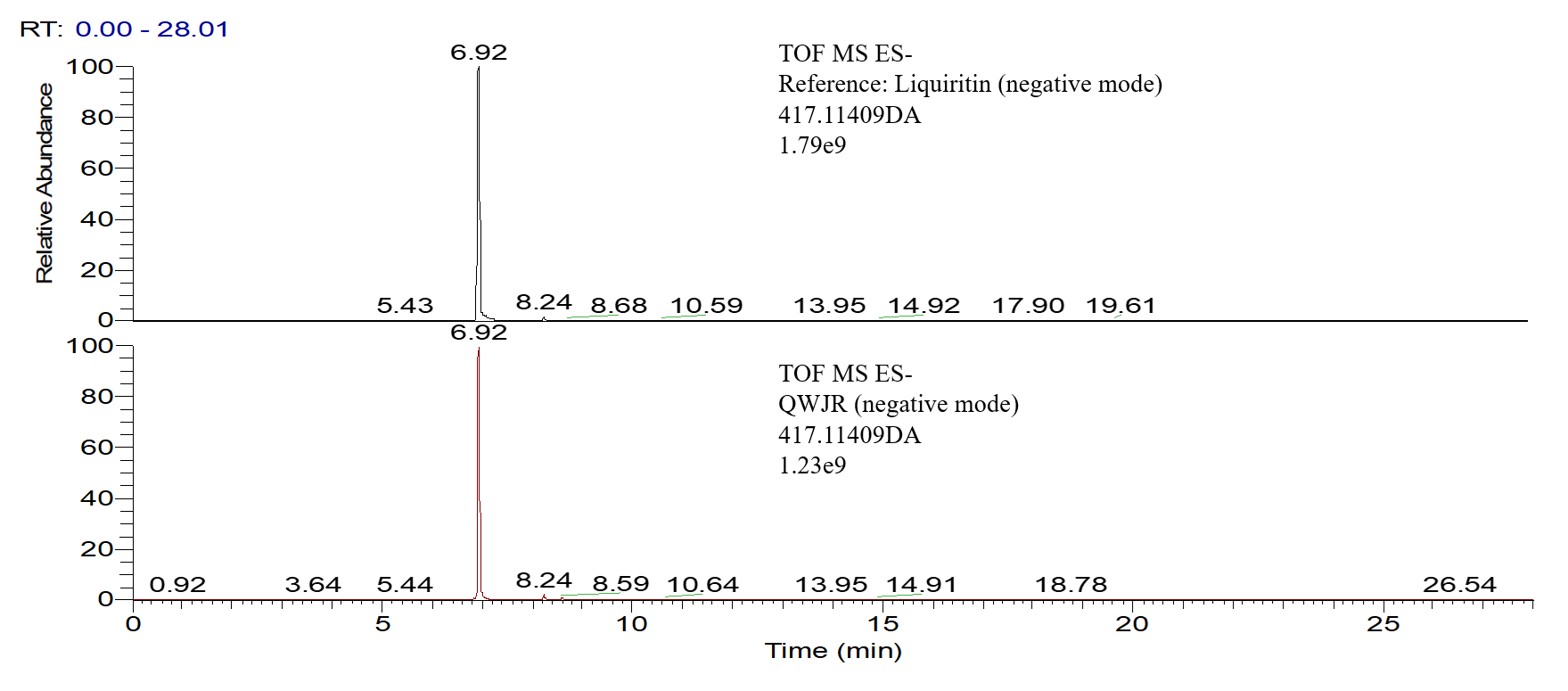
**

**F**

**
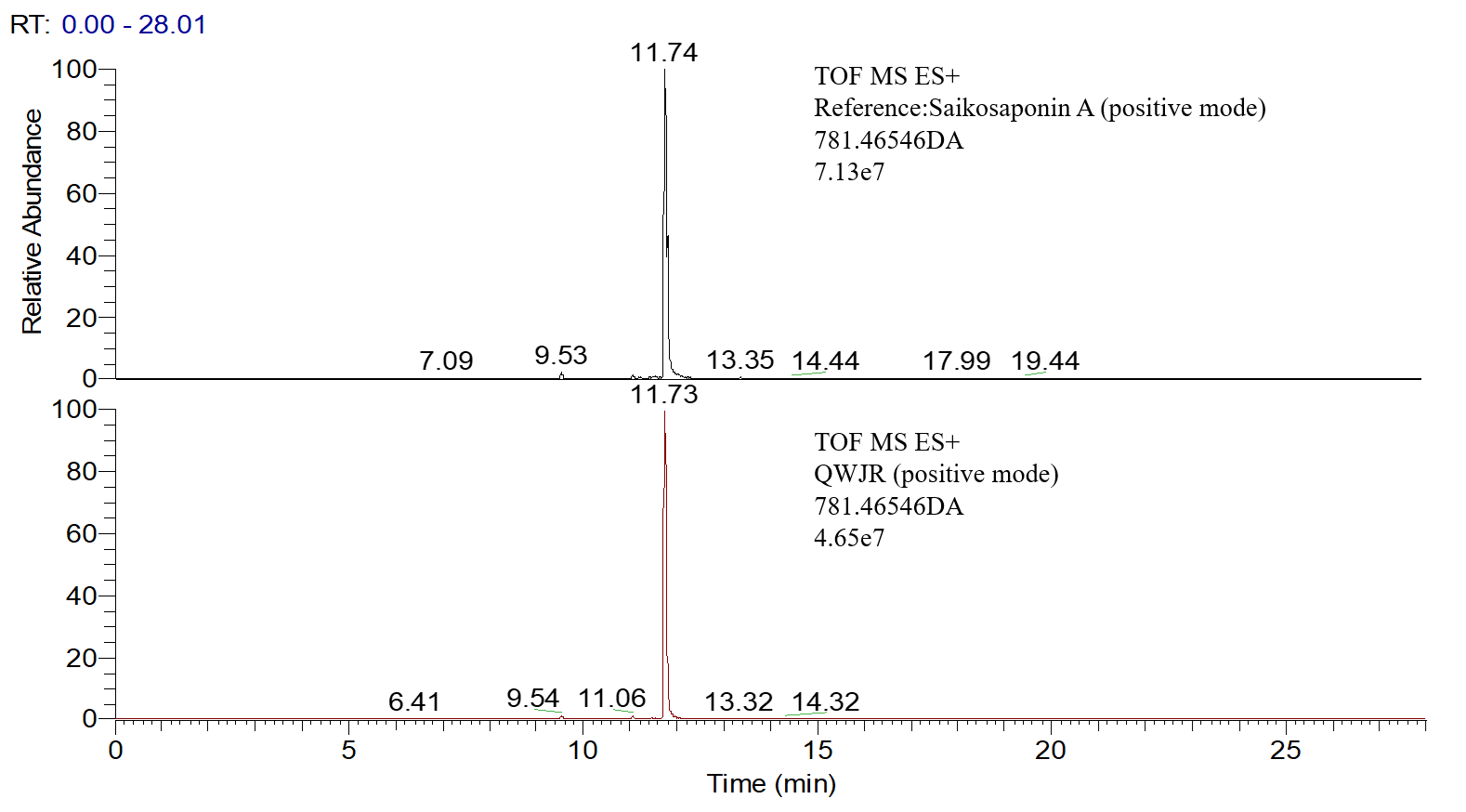
**

**G**

**
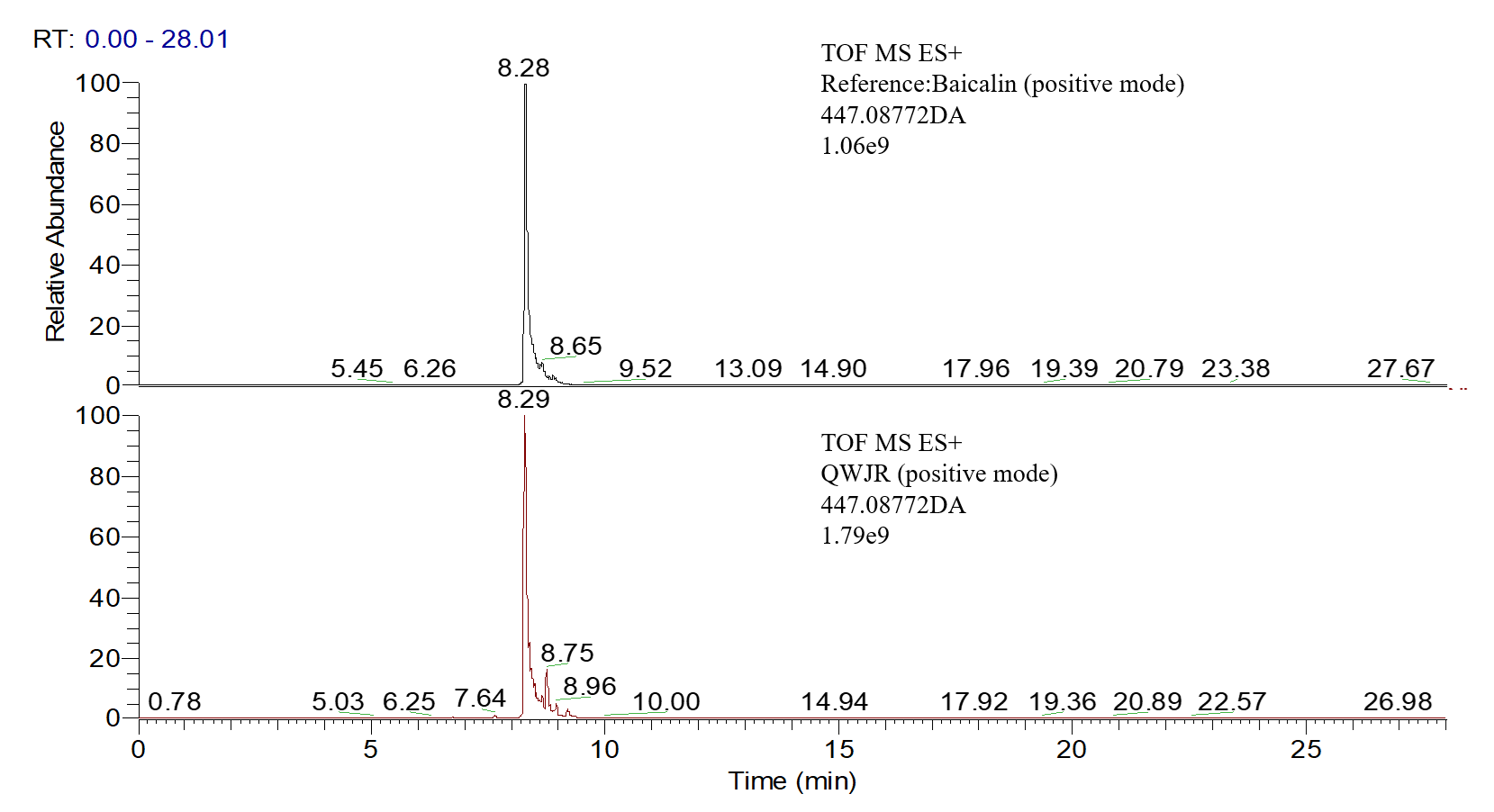
**

**H**

**
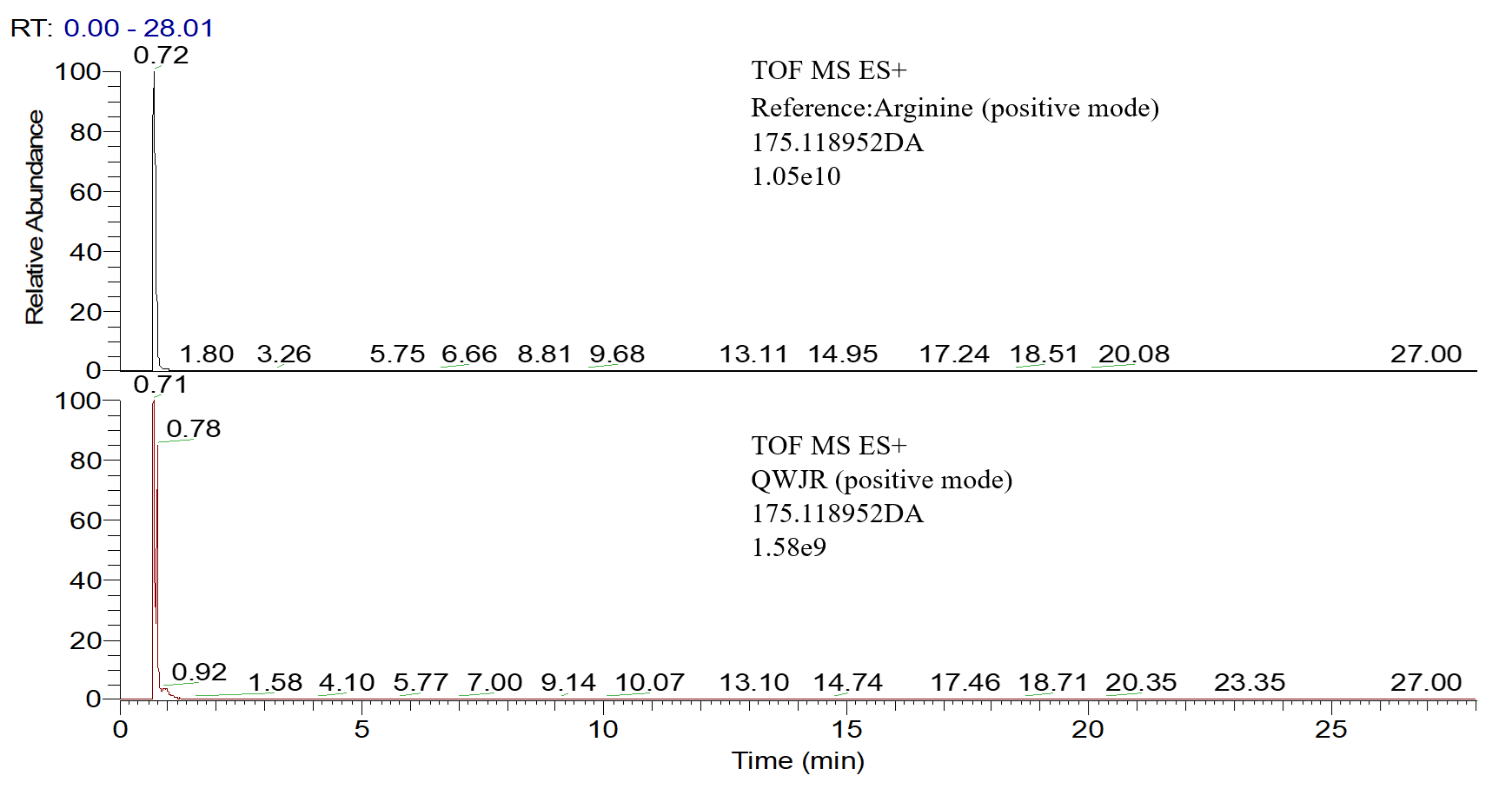
**

**I**

**
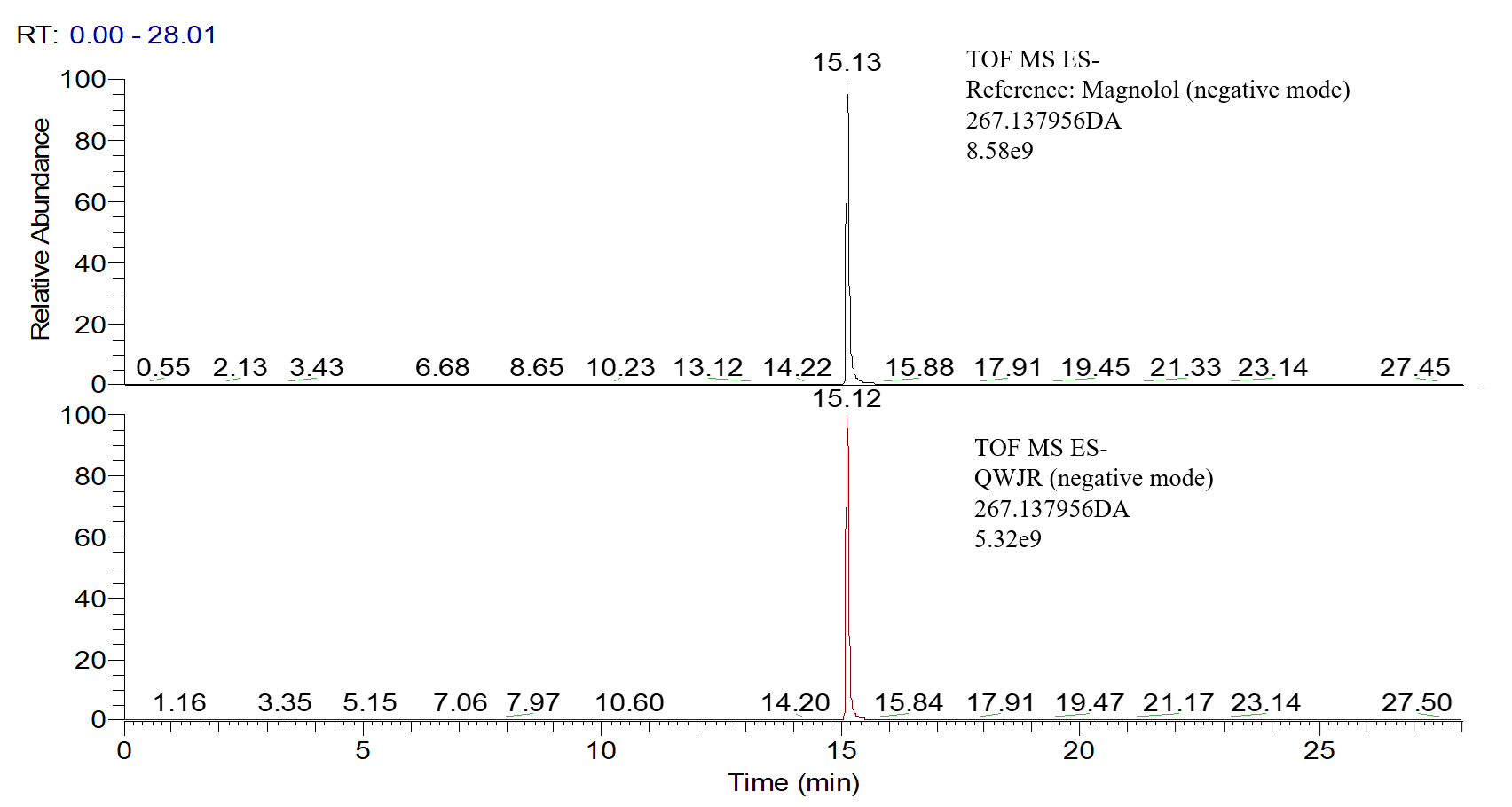
**

**J**


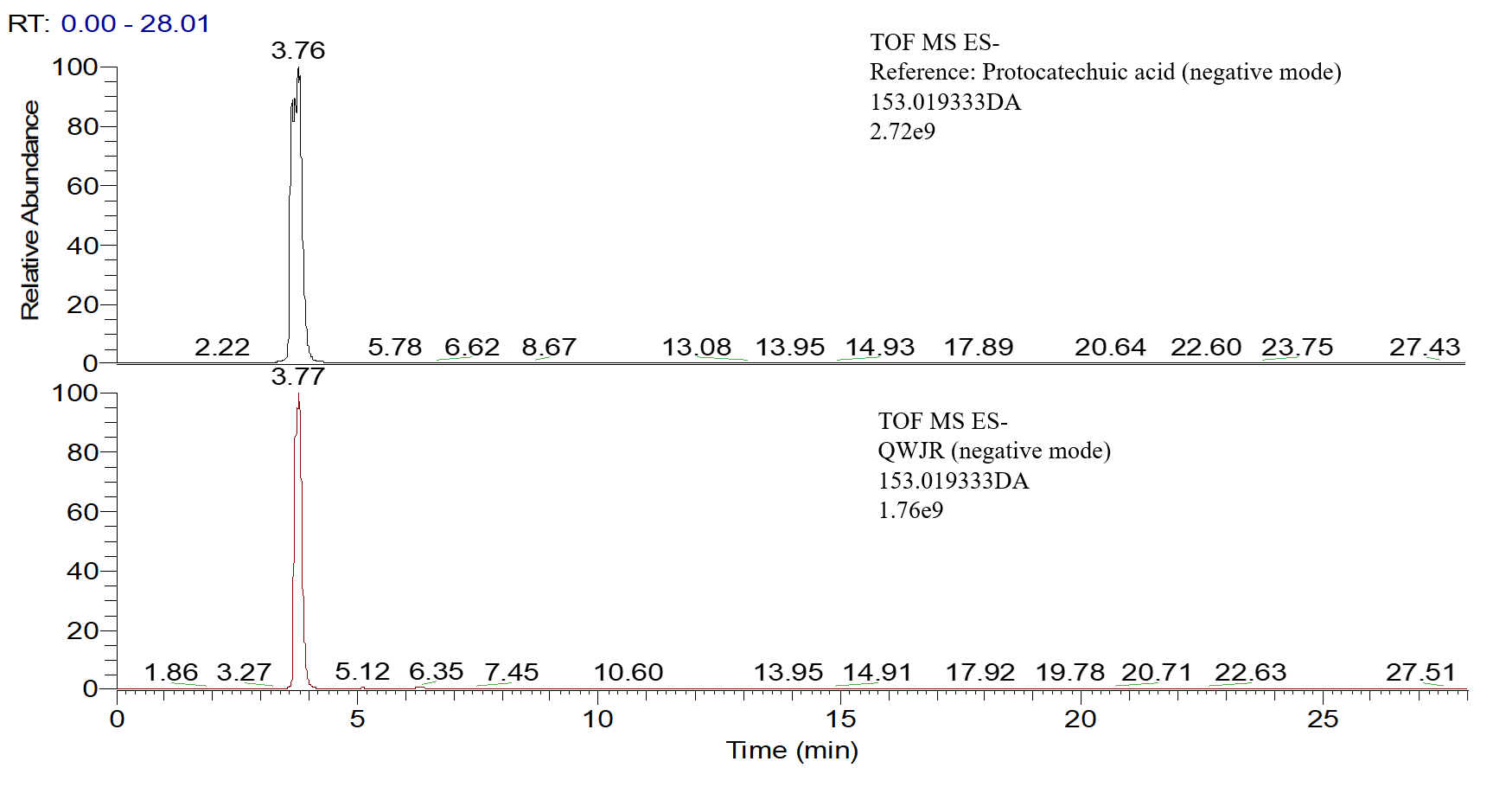


**K**

**
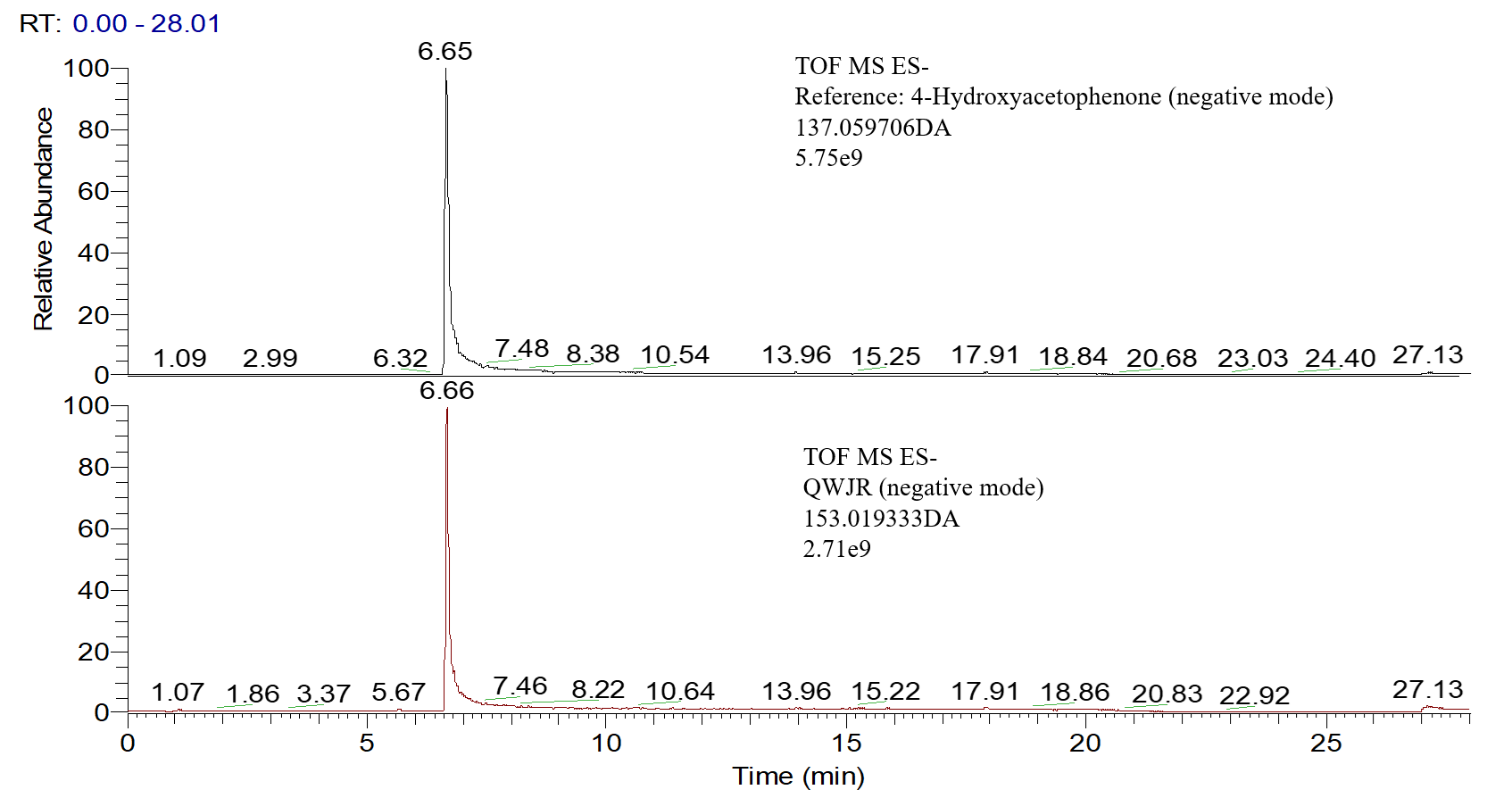
**

**L**

**
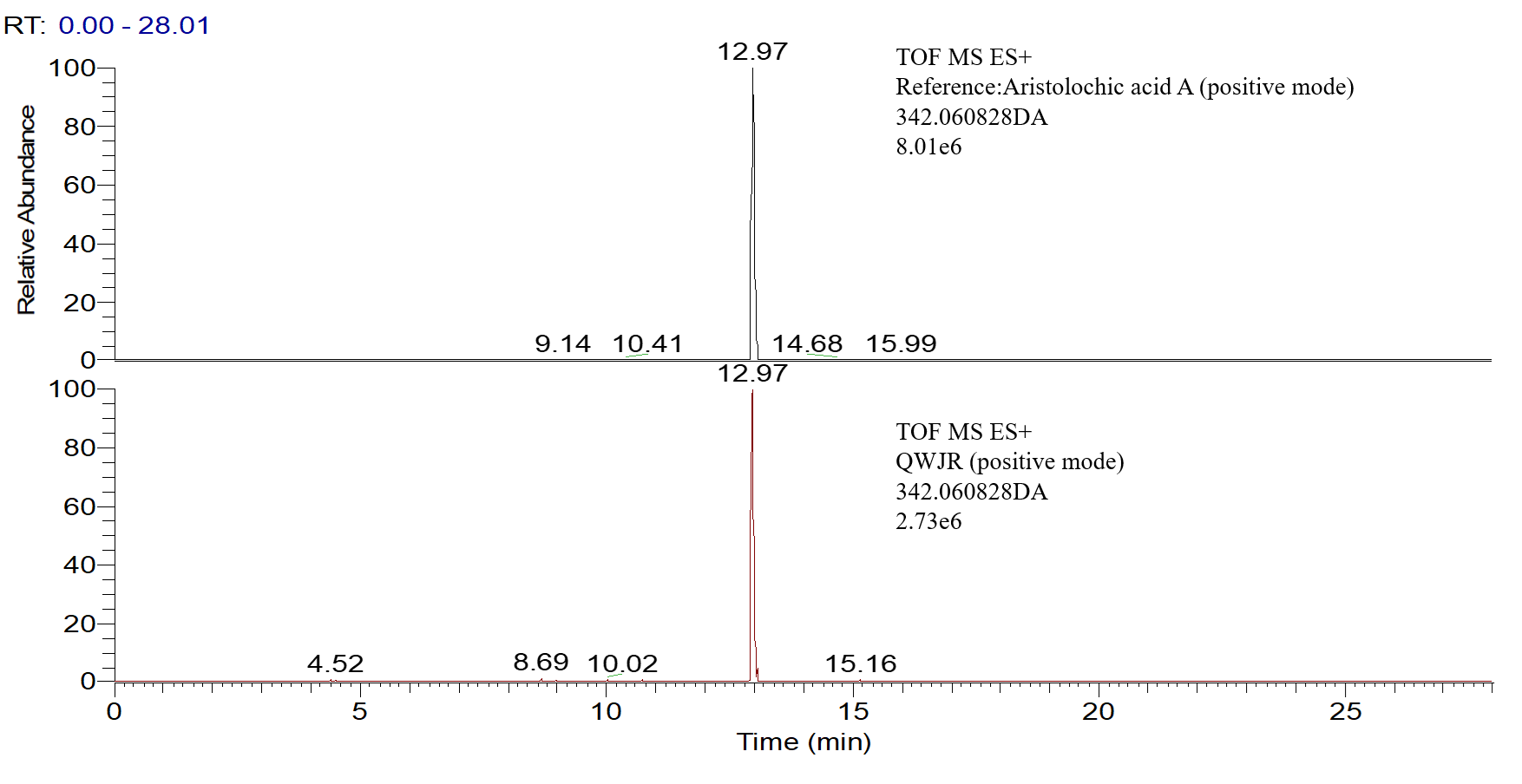
**

**M**

**
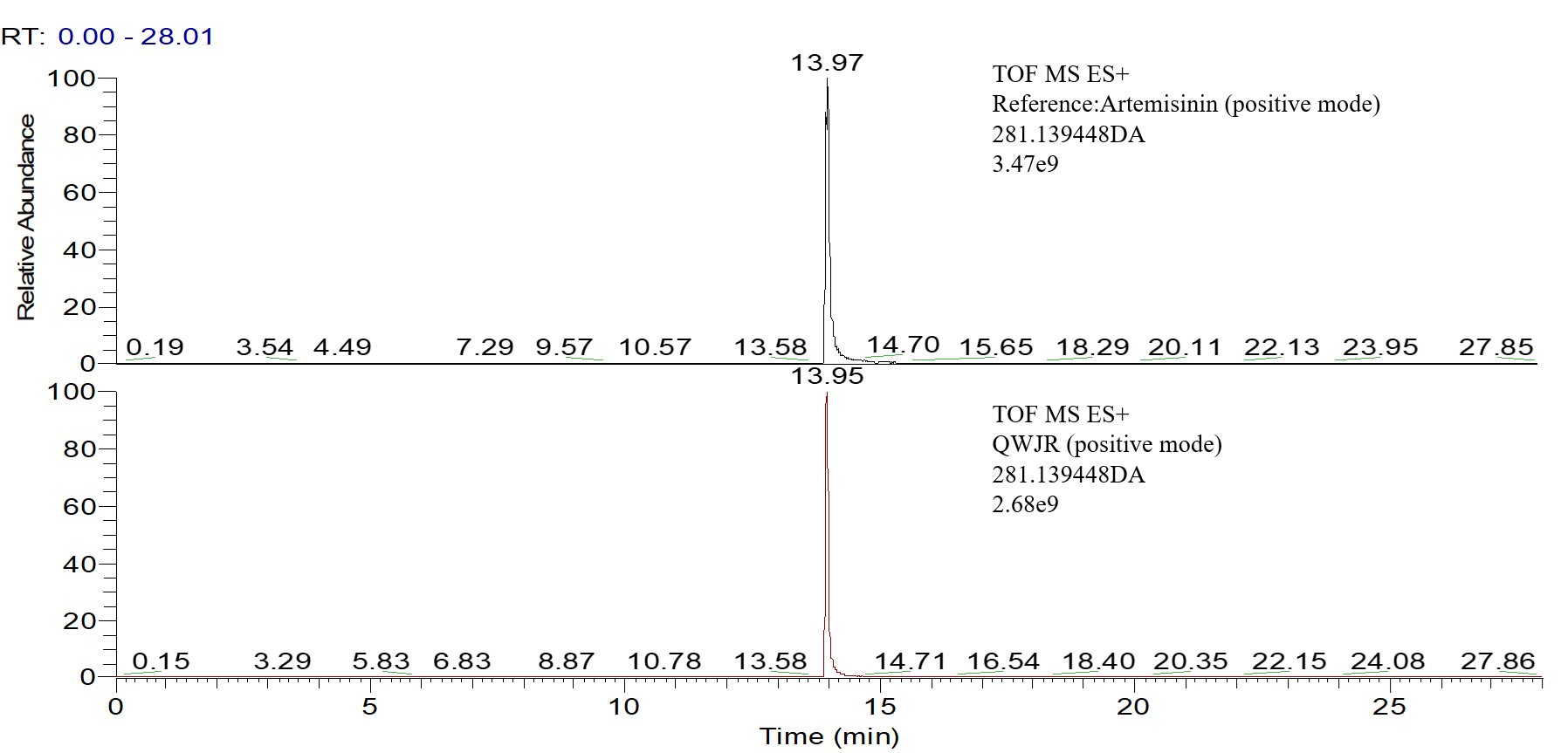
**

**FIGURE S2:** The chemical profiles of QWJR using UPLC-MS. (**A, B**) The total ion chromatogram in positive (**A**) and negative ion modes (**B**). (**C-L**) The main bioactive compounds of Chlorogenic acid (**C**), Forsythoside A (**D**), Liquiritin (**E**), Saikosaponin A (**F**), Baicalin (**G**), Arginine (**H**), Magnolol (**I**), Protocatechuic acid (**J**), 4-Hydroxyacetophenone (**K**), Aristolochic acid A (**L**), Artemisinin (**M**). The bioactive compounds detected in QWJR were confirmed by the reference standards.

**TABLE S1 The characteristic fragment ions of reference standards in QWJR**

| **Marking**  **peak no.** | **Name** | **RT**  **(min)** | **Ion** |
| --- | --- | --- | --- |
| 1 | Chlorogenic acid | 5.16 | [M+H]^+^ |
| 2 | Forsythoside A | 6.64 | [M-H]^+^ |
| 3 | Liquiritin | 6.92 | [M+H]^-^ |
| 4 | Saikosaponin A | 11.73 | [M-H]^+^ |
| 5 | Baicalin | 8.29 | [M-H]^+^ |
| 6 | Arginine | 0.71 | [M-H]^+^ |
| 7 | Magnolol | 15.13 | [M+H]^-^ |
| 8 | Protocatechuic acid | 3.77 | [M-H]^-^ |
| 9 | 4-Hydroxyacetophenone | 6.66 | [M-H]^+^ |
| 10 | Aristolochic acid A | 12.97 | [M-H]^+^ |
| 11 | Artemisinin | 13.95 | [M+H]^+^ |
