# Supplementary material for: Qing-Wen-Jie-Re Mixture Ameliorates Poly (I:C)-Induced Viral Pneumonia Through Regulating the Inflammatory Response and Serum Metabolism
Source: Front Pharmacol. 2022 Jun 15;13:891851. doi: 10.3389/fphar.2022.891851 (PMC9240632; doi:10.3389/fphar.2022.891851)
Supplement: Supplementary file 2 [file Table2.DOCX]

**TABLE S2 The metabolites varied in both the M vs C and Q vs M groups**

| **No.** | **Formula** | **RT [min]** | **m/z** | **Metabolites** | **HMDB ID** | **VIP** | | | **FC** | | | **Trend** | | |
| --- | --- | --- | --- | --- | --- | --- | --- | --- | --- | --- | --- | --- | --- | --- |
|  |  |  |  |  |  | **M vs. C** | **Q vs. M** | **Q vs.C** | **M vs. C** | **Q vs. M** | **Q vs.C** | **M vs. C** | **Q vs. M** | **Q vs.C** |
| 1 | C_9_H_9_NO_3_ | 5.49 | 178.05 | Hippuric acid | HMDB0000714 | 1.41 | 1.44 | 1.40 | 0.41 | 1.72 | 1.74 | ↓^##^ | ↑^**^ | ↑^##^ |
| 2 | C_21_H_30_O_4_ | 7.53 | 345.21 | Cortodoxone | HMDB0000015 | 1.78 | 1.70 | 1.48 | 0.64 | 1.45 | 0.65 | ↓^##^ | ↑^**^ | ↓^##^ |
| **3** | **C_6_H_6_O_6_** | **1.45** | **173.01** | **cis-Aconitic acid** | **HMDB0000072** | **1.29** | **1.68** | **0.18** | **0.25** | **3.55** | **1.02** | **↓^##^** | **↑^**^** | **—** |
| **4** | **C_11_H_12_N_2_O_2_** | **6.79** | **203.08** | **L-Tryptophan** | **HMDB0000929** | **1.73** | **1.08** | **0.16** | **0.41** | **2.72** | **1.01** | **↓^##^** | **↑^*^** | **—** |
| 5 | C_6_H_8_O_6_ | 1.45 | 175.02 | Ascorbic acid | HMDB0000044 | 1.48 | 1.89 | 1.80 | 8.43 | 0.61 | 2.28 | ↑^#^ | ↓^*^ | ↑^#^ |
| 6 | C_26_H_43_NO_6_ | 7.21 | 464.30 | Glycocholic acid | HMDB0000138 | 1.34 | 1.03 | 1.04 | 8.58 | 2.99 | 3.42 | ↑^#^ | ↑^*^ | ↑^##^ |
| **7** | **C_6_H_13_N_3_O_3_** | **1.38** | **174.09** | **Citrulline** | **HMDB0000904** | **1.58** | **1.21** | **0.88** | **0.59** | **1.67** | **1.01** | **↓^##^** | **↑^*^** | **—** |
| 8 | C_7_H_11_N_3_O_2_ | 1.33 | 168.08 | 3-Methylhistidine | HMDB0000479 | 1.63 | 1.44 | 1.13 | 0.29 | 0.45 | 0.35 | ↓^##^ | ↓^*^ | ↓^##^ |
| **9** | **C_5_H_6_O_5_** | **1.55** | **145.01** | **alpha-Ketoglutaric acid** | **HMDB0000208** | **1.62** | **1.96** | **0.37** | **0.39** | **1.78** | **1.00** | **↓^##^** | **↑^*^** | **—** |
| 10 | C_10_H_12_N_4_O_6_ | 4.79 | 283.07 | Xanthosine | HMDB0000299 | 1.53 | 1.20 | 1.18 | 2.67 | 0.75 | 0.61 | ↑^#^ | ↓^*^ | ↓^#^ |
| 11 | C_27_H_44_O_3_ | 9.94 | 415.32 | Calcitriol | HMDB0001903 | 1.35 | 1.62 | 0.86 | 2.87 | 1.45 | 2.30 | ↑^#^ | **↑**^*^ | ↑^##^ |
| 12 | C_24_H_40_O_4_ | 7.41 | 391.29 | Deoxycholic acid | HMDB0000626 | 1.50 | 1.37 | 0.30 | 4.76 | 2.99 | 1.45 | ↑^#^ | ↑^*^ | **↑**^##^ |
| **13** | **C_22_H_32_O_2_** | **10.09** | **327.23** | **Docosahexaenoic acid** | **HMDB0002183** | **1.44** | **1.05** | **0.72** | **1.54** | **0.72** | **1.01** | **↑^#^** | **↓^**^** | **—** |
| **14** | **C_12_H_18_O_3_** | **6.43** | **209.12** | **Jasmonic acid** | **HMDB0032797** | **1.37** | **1.34** | **0.27** | **0.41** | **1.46** | **0.99** | **↓^##^** | **↑^*^** | **—** |
| 15 | C_11_H_13_NO_3_ | 5.82 | 206.08 | N-Acetyl-L-phenylalanine | HMDB0000512 | 1.48 | 1.45 | 1.41 | 2.64 | 0.68 | 1.73 | ↑^#^ | ↓^**^ | ↑^##^ |
| 16 | C_10_H_10_O_4_ | 5.42 | 193.05 | Ferulic acid | HMDB0000954 | 1.43 | 1.48 | 0.58 | 4.24 | 2.19 | 2.89 | ↑^#^ | ↑^*^ | ↑^##^ |
| **17** | **C_20_H_32_O_5_** | **8.18** | **333.21** | **Prostaglandin H2** | **HMDB0001381** | **1.43** | **1.73** | **0.06** | **0.49** | **6.82** | **1.01** | **↓^#^** | **↑^**^** | **—** |
| 18 | C_6_H_10_O_6_ | 1.41 | 177.04 | Gluconolactone | HMDB0000150 | 1.15 | 1.95 | 1.64 | 0.64 | 1.37 | 3.86 | ↓^##^ | ↑^**^ | ↑^##^ |
| 19 | C_24_H_40_O_5_ | 7.12 | 407.28 | Cholic acid | HMDB0000619 | 1.07 | 1.58 | 1.48 | 0.76 | 0.79 | 0.71 | ↓^##^ | ↓^*^ | ↓^#^ |
| 20 | C_24_H_40_O_4_ | 8.40 | 437.29 | Chenodeoxycholic Acid | HMDB0000518 | 1.26 | 1.12 | 1.71 | 4.93 | 1.26 | 1.65 | ↑^#^ | ↑^*^ | ↑^##^ |
| **21** | **C_6_H_8_O_7_** | **1.55** | **191.02** | **Citric acid** | **HMDB0000094** | **1.65** | **1.17** | **0.05** | **2.13** | **0.76** | **0.98** | **↑^##^** | **↓^*^** | **—** |
| **22** | **C_33_H_36_N_4_O_6_** | **7.55** | **585.27** | **Bilirubin** | **HMDB0000054** | **1.66** | **1.24** | **0.11** | **2.81** | **0.27** | **1.00** | **↑^##^** | **↓^*^** | **—** |
| 23 | C_11_H_12_N_2_O_2_ | 6.81 | 205.10 | Tryptophan | HMDB0000929 | 1.79 | 1.34 | 1.49 | 0.28 | 1.45 | 0.69 | ↓^##^ | ↑^**^ | ↓^##^ |
| **24** | **C_28_H_44_O** | **8.00** | **397.34** | **Ergocalciferol** | **HMDB0000900** | **1.86** | **1.18** | **0.66** | **0.22** | **1.83** | **1.01** | **↓^##^** | **↑^**^** | **—** |
| 25 | C_4_H_7_N_3_O | 1.42 | 136.05 | Creatinine | HMDB0000562 | 1.73 | 1.58 | 0.44 | 0.57 | 0.74 | 0.76 | ↓^##^ | ↓^*^ | ↓^##^ |
| 26 | C_18_H_24_O_3_ | 7.45 | 311.16 | Estriol | HMDB0000347 | 1.84 | 1.13 | 1.85 | 0.24 | 0.43 | 0.18 | ↓^##^ | ↓^*^ | ↓^##^ |
| 27 | C_19_H_24_O_3_ | 7.28 | 301.18 | Adrenosterone | HMDB0006772 | 1.85 | 1.45 | 1.32 | 0.16 | 2.25 | 0.28 | ↓^##^ | ↑^**^ | ↓^##^ |
| 28 | C_21_H_28_O_5_ | 5.81 | 721.39 | Cortisone | HMDB0002802 | 1.70 | 1.20 | 0.58 | 0.52 | 0.46 | 0.68 | ↓^##^ | ↓^*^ | ↓^##^ |
| **29** | **C_6_H_6_N_2_O** | **2.01** | **123.06** | **Nicotinamide** | **HMDB0001406** | **1.47** | **1.34** | **0.39** | **2.11** | **0.21** | **1.00** | ↑**^#^** | ↓**^*^** | **—** |
| 30 | C_10_H_11_NO_3_ | 5.64 | 194.08 | Phenylacetylglycine | HMDB0000821 | 1.18 | 1.30 | 1.14 | 3.86 | 0.58 | 0.80 | ↑^#^ | ↓^*^ | ↓^##^ |
| **31** | **C_5_H_12_N_2_O_2_** | **1.19** | **133.10** | **L-Ornithine** | **HMDB0000214** | **1.60** | **1.44** | **0.24** | **0.33** | **1.45** | **1.00** | **↓^##^** | **↑**** | **—** |
| **32** | **C_7_H_8_N_2_O** | **1.39** | **137.07** | **1-Methylnicotinamide** | **HMDB0000699** | **1.44** | **1.27** | **0.86** | **2.60** | **0.37** | **0.98** | **↑^#^** | **↓^*^** | **—** |
| 33 | C_10_H_16_N_4_O_3_ | 1.34 | 241.13 | Anserine | HMDB0000194 | 1.52 | 1.58 | 0.82 | 0.13 | 0.62 | 1.56 | ↓^##^ | ↓^*^ | ↑^##^ |
| 34 | C_18_H_30_O_3_ | 8.56 | 295.23 | 13(S)-HOTrE | -- | 1.18 | 1.10 | 1.07 | 0.36 | 0.48 | 0.81 | ↓^##^ | ↓^*^ | ↓^#^ |
| **35** | **C_33_H_46_N_4_O_6_** | **5.78** | **595.35** | **Stercobilin** | **HMDB0004159** | **1.08** | **2.05** | **0.34** | **0.16** | **3.73** | **1.00** | **↓^#^** | **↑^**^** | **—** |
| 36 | C_16_H_30_O_2_ | 8.02 | 277.22 | Palmitoleic Acid | HMDB0012328 | 1.19 | 1.20 | 1.94 | 0.44 | 5.01 | 1.35 | ↓^##^ | ↑^**^ | ↑^##^ |
| 37 | C_19_H_28_O_3_ | 8.73 | 322.24 | 16α-Hydroxydehydroepiandrosterone | -- | 1.28 | 1.85 | 1.85 | 0.56 | 0.76 | 0.48 | ↓^##^ | ↓^*^ | ↓^#^ |
| **38** | **C_5_H_11_NO_2_** | **1.39** | **118.09** | **Betaine** | **HMDB0000043** | **1.06** | **1.53** | **0.26** | **0.65** | **1.54** | **1.04** | **↓^#^** | **↑^**^** | **—** |
| **39** | **C_18_H_22_O_2_** | **6.98** | **253.16** | **Estrone** | **HMDB0000145** | **1.03** | **1.28** | **0.68** | **2.11** | **0.20** | **1.00** | **↑^##^** | **↓^*^** | **—** |
| 40 | C_7_H_6_O_3_ | 6.21 | 137.02 | 4-Hydroxybenzoic acid | HMDB0000500 | 1.21 | 2.31 | 1.56 | 0.49 | 2.80 | 1.91 | ↓^##^ | ↑^**^ | ↑^#^ |
| 41 | C_24_H_40_O_3_ | 9.65 | 375.29 | Lithocholic acid | HMDB0000761 | 1.38 | 2.21 | 0.36 | 1.45 | 1.40 | 1.42 | ↑^#^ | ↑^*^ | ↑^##^ |
| 42 | C_9_H_17_NO_4_ | 5.88 | 202.11 | O-Acetyl-L-carnitine | HMDB0000201 | 1.40 | 2.09 | 1.92 | 0.69 | 4.06 | 0.10 | ↓^##^ | ↑^**^ | ↓^#^ |
| 43 | C_7_H_8_O | 5.84 | 107.05 | 4-Methylphenol | HMDB0001858 | 1.46 | 1.45 | 1.69 | 1.45 | 1.73 | 0.73 | ↑^#^ | ↑^*^ | ↓^#^ |
| **44** | **C_20_H_32_O_6_** | **7.24** | **367.21** | **Prostaglandin G2** | **HMDB0003235** | **1.59** | **2.21** | **0.73** | **1.88** | **0.22** | **0.99** | **↑^#^** | **↓^**^** | **—** |
| 45 | C_22_H_36_O_2_ | 10.82 | 331.26 | Adrenic acid | HMDB0002226 | 1.96 | 1.45 | 1.50 | 0.21 | 0.50 | 0.46 | ↓^##^ | ↓^*^ | ↓^##^ |
| 46 | C_15_H_12_I_3_NO_4_ | 5.43 | 649.78 | Triiodothyronine | HMDB0000265 | 1.74 | 1.96 | 0.99 | 0.56 | 0.47 | 0.56 | ↓^##^ | ↓^*^ | ↓^#^ |
| 47 | C_2_H_7_NO_3_S | 1.52 | 124.01 | Taurine | HMDB0000251 | 1.41 | 1.57 | 1.01 | 1.47 | 0.65 | 1.39 | ↑^##^ | ↓^*^ | ↑^#^ |
| 48 | C_5_H_4_N_2_O_4_ | 1.47 | 155.01 | Orotic Acid | HMDB0000226 | 1.72 | 1.45 | 0.75 | 1.86 | 1.53 | 1.47 | ↑^#^ | ↑^*^ | ↑^##^ |
| 49 | C_22_H_34_O_2_ | 10.38 | 329.25 | Docosapentaenoic acid | HMDB0001976 | 1.25 | 1.84 | 1.36 | 0.13 | 0.62 | 0.87 | ↓^##^ | ↓^*^ | ↓^##^ |
| 50 | C_43_H_58_N_4_O_12_ | 7.19 | 821.40 | Rifampicin | -- | 1.29 | 1.55 | 1.42 | 0.78 | 0.19 | 0.69 | ↓^##^ | ↓^*^ | ↓^##^ |
| 51 | C_15_H_11_I_4_ NO_4_ | 6.56 | 775.68 | L-Thyroxine | HMDB0000248 | 1.63 | 1.71 | 1.43 | 1.75 | 0.52 | 0.65 | ↑^##^ | ↓^**^ | ↓^##^ |
| 52 | C_5_H_4_N_4_O_3_ | 2.00 | 167.02 | Uric acid | HMDB0000289 | 1.95 | 1.13 | 1.67 | 0.32 | 0.65 | 0.28 | ↓^##^ | ↓^*^ | ↓^##^ |
| **53** | **C_20_H_32_O_5_** | **6.59** | **333.21** | **Prostaglandin D2** | **HMDB0001403** | **1.37** | **1.42** | **0.15** | **0.60** | **3.16** | **1.01** | **↓^#^** | **↑^**^** | **—** |
| 54 | C_17_H_21_N_4_O_9_P | 5.99 | 455.10 | Riboflavin-5-phosphate | HMDB0001520 | 1.00 | 1.05 | 0.23 | 0.79 | 0.55 | 0.65 | ↓^##^ | ↓^*^ | ↓^##^ |
| 55 | C_19_H_30_O_2_ | 9.89 | 289.22 | Androsterone | HMDB0000031 | 1.75 | 1.20 | 1.41 | 0.21 | 2.65 | 0.61 | ↓^#^ | ↑^**^ | ↓^##^ |
| 56 | C_15_H_12_O_5_ | 6.17 | 271.06 | Naringenin | HMDB0002670 | 1.99 | 1.35 | 0.14 | 1.46 | 3.70 | 1.80 | ↑^#^ | ↑^*^ | ↑^##^ |
| 57 | C_20_H_30_O_2_ | 9.09 | 285.22 | Eicosapentaenoic acid | HMDB0001999 | 1.44 | 1.20 | 0.52 | 4.30 | 1.51 | 0.71 | ↑^#^ | ↑^*^ | ↓^##^ |
| **58** | **C_20_H_32_O_3_** | **8.56** | **343.22** | **16(R)-HETE** | **HMDB0004680** | **1.40** | **1.90** | **0.48** | **0.47** | **2.56** | **1.00** | **↓^#^** | **↑^**^** | **—** |
| 59 | C_12_H_23_O_14_P | 1.20 | 423.09 | Trehalose 6-phosphate | HMDB0001124 | 1.76 | 1.49 | 1.49 | 0.76 | 0.79 | 0.48 | ↓^#^ | ↓^**^ | ↓^##^ |
| 60 | C_21_H_30_O_3_ | 7.47 | 331.23 | Deoxycorticosterone | HMDB0000016 | 1.52 | 1.30 | 1.60 | 0.89 | 2.63 | 0.38 | ↓^##^ | ↑^**^ | ↓^##^ |
| 61 | C_21_H_32_O_5_ | 7.23 | 347.22 | Tetrahydrocortisone | HMDB0000903 | 1.47 | 1.97 | 1.17 | 0.45 | 4.31 | 0.28 | ↓^##^ | ↑^*^ | ↓^##^ |
| 62 | C_21_H_30_O_5_ | 6.89 | 363.22 | Hydrocortisone | HMDB0000063 | 1.71 | 1.38 | 1.19 | 2.12 | 1.93 | 2.52 | ↑^#^ | ↑^*^ | ↑^##^ |
| **63** | **C_18_H_30_O_3_** | **8.24** | **317.21** | **13-OxoODE** | **HMDB0004668** | **1.86** | **1.23** | **0.10** | **0.44** | **1.62** | **1.00** | **↓**^##^ | **↑^*^** | **—** |
| **64** | **C_18_H_16_O_8_** | **5.11** | **356.13** | **Rosmarinic acid** | **HMDB0003572** | **1.61** | **1.25** | **0.74** | **1.60** | **0.76** | **1.01** | **↑^#^** | ↓**^**^** | **—** |
| 65 | C_21_H_30_O_2_ | 7.66 | 315.23 | Progesterone | HMDB0001830 | 1.31 | 1.38 | 1.24 | 0.31 | 0.62 | 0.71 | ↓^##^ | ↓^*^ | ↓^#^ |
| 66 | C_33_H_34_N_4_O_6_ | 6.61 | 583.26 | Biliverdin | HMDB0001008 | 1.97 | 1.07 | 1.40 | 0.53 | 1.52 | 3.13 | ↓^##^ | ↑^*^ | ↑^##^ |

Control, model and QWJR-H (*n* = 6 per group) groups.

^#^: *p* < 0.05 as compared to the control group; ^##^: *p* < 0.01 as compared to the control group; ^*^: *p* < 0.05 as compared to the model group; ^**^: *p* < 0.01 as compared to the model group; ↑: content increased (FC > 1.25); ↓: content decreased (FC < 0.8); **—:** content doesn't change much

(FC≈1); vs: versus; C: control group; M: model group; Q: QWJR-H group.
